# Comparative transcriptome analyses reveal different mechanism of high- and low-tillering genotypes controlling tiller growth in orchardgrass (*Dactylis glomerata* L.)

**DOI:** 10.1186/s12870-020-02582-2

**Published:** 2020-08-05

**Authors:** Xiaoheng Xu, Guangyan Feng, Yueyang Liang, Yang Shuai, Qiuxu Liu, Gang Nie, Zhongfu Yang, Linkai Hang, Xinquan Zhang

**Affiliations:** 1 Department of Grassland Science, College of Animal Science and Technology, Sichuan Agricultural University, Chengdu, China; 2 Rice Research Institute, Sichuan Agricultural University, Chengdu, China

**Keywords:** Molecular mechanisms, Orchardgrass, Plant hormones, RNA-seq, Tillering regulation

## Abstract

**Background:**

Tillering is an important agronomic trait underlying the yields and reproduction of orchardgrass (*Dactylis glomerata*), an important perennial forage grass. Although some genes affecting tiller initiation have been identified, the tillering regulatory network is still largely unknown, especially in perennial forage grasses. Thus, unraveling the regulatory mechanisms of tillering in orchardgrass could be helpful in developing selective strategies for high-yield perennial grasses. In this study, we generated high-throughput RNA-sequencing data from multiple tissues of tillering stage plants to identify differentially expressed genes (DEGs) between high- and low-tillering orchardgrass genotypes. Gene Ontology and pathway enrichment analyses connecting the DEGs to tillering number diversity were conducted.

**Results:**

In the present study, approximately 26,282 DEGs were identified between two orchardgrass genotypes, AKZ-NRGR667 (a high-tillering genotype) and D20170203 (a low-tillering genotype), which significantly differed in tiller number. Pathway enrichment analysis indicated that DEGs related to the biosynthesis of three classes of phytohormones, i.e., strigolactones (SLs), abscisic acid (ABA), and gibberellic acid (GA), as well as nitrogen metabolism dominated such differences between the high- and low-tillering genotypes. We also confirmed that under phosphorus deficiency, the expression level of the major SL biosynthesis genes encoding DWARF27 (D27), 9-cis-beta-carotene 9′,10′-cleaving dioxygenase (CCD7), carlactone synthase (CCD8), and more axillary branching1 (MAX1) proteins in the high-tillering orchardgrass genotype increased more slowly relative to the low-tillering genotype.

**Conclusions:**

Here, we used transcriptomic data to study the tillering mechanism of perennial forage grasses. We demonstrated that differential expression patterns of genes involved in SL, ABA, and GA biosynthesis may differentiate high- and low-tillering orchardgrass genotypes at the tillering stage. Furthermore, the core SL biosynthesis-associated genes in high-tillering orchardgrass were more insensitive than the low-tillering genotype to phosphorus deficiency which can lead to increases in SL biosynthesis, raising the possibility that there may be distinct SL biosynthesis way in tillering regulation in orchardgrass. Our research has revealed some candidate genes involved in the regulation of tillering in perennial grasses that is available for establishment of new breeding resources for high-yield perennial grasses and will serve as a new resource for future studies into molecular mechanism of tillering regulation in orchardgrass.

## Background

Tillering is a specialized branching in grasses that occurs from the basal node and grows independently of the mother stem by means of its own adventitious roots [1, 2]. More importantly, tillering is an important agronomic characteristic of forage grasses which ultimately determines the aboveground biomass and seed yield of the species [3, 4]. Orchardgrass (*Dactylis glomerata* L.) is a winter perennial grass native to northern Africa, western and central Europe, and temperate Asia [5]. As an important perennial forage grass, orchardgrass has been widely cultivated for a long time owing to its high adaptability, nutritive value, and biomass [6]. In China, orchardgrass is not only an excellent forage grass, but also one of the important mixed grasses in artificial grasslands [7]. However, as a perennial forage grass, the sexual and asexual reproduction of orchardgrass both occur directly through tillering [4, 8]. Therefore, tillering is of great significance to the yield and reproduction of orchardgrass. With the publication of the orchardgrass genome [9], unraveling the regulatory mechanisms that underly tillering in orchardgrass can be used to develop selective breeding resources for high-yield perennial grasses.

Tillering is a very complex process influenced by many factors, including nutrients, hormones, and genetic factors. Among these factors, nitrogen has a particularly substantial regulatory effect on tillering in rice, both promoting the initiation and outgrowth of tiller buds [10]. Moreover, the outgrowth of lateral shoots can be inhibited by gibberellic acid (GA), abscisic acid (ABA), or strigolactones (SLs) [11–15], while bud outgrowth can be promoted by cytokinins (CKs) and brassinosteroids (BRs) [16]. Recently, it has been reported that ABA and GA can promote tiller formation by suppressing SL biosynthesis [17, 18]. Although many studies have shown that tiller initiation can be regulated by the coordinated action of plant hormones rather than a single hormone [19], there has been no applicable model explaining the relationship between a variety of hormones and the initiation of tillers because of the complexity of plant hormones regulating lateral bud growth [13]. However, some genes affecting tiller initiation have been identified, and they fall into two main groups. One group consisting of the four key tiller formation regulators has been cloned, including *MONOCULM 1* (*MOC1*), *TILLERS ABSENT 1*/*STERILE AND REDUCED TILLERING 1 (MOC3*/*TAB1*/*SRT1*), *LAX PANICLE 1* (*LAX1*), and *LAX2,* which control the formation of tiller buds [2, 20–24]. Moreover, MOC1 and MOC3 have been reported to interact with each other to regulate the outgrowth of tiller buds by upregulating the expression of *FLORAL ORGAN NUMBER1* (*FON1*) in rice [25]. The other group contains genes that are associated with the biosynthesis and signal transduction of phytohormones, including *gibberellin 2beta-dioxygenase* (*GA2ox*), *DWARF27* (*D27*), and *DWARF53* (*D53*), which control the outgrowth of tiller buds [11, 26, 27]. For example, Lin et al. reported that *D27* is involved in the biosynthesis of strigolactones, which regulate the outgrowth of tiller buds in rice [26]. Moreover, SLs have been reported to induce D53 degradation by the proteasome and abrogate the activity of D53 in promoting axillary bud outgrowth in a DWARF14 (D14)- and DWARF3 (D3)-dependent manner [27, 28]. However, the regulatory network underlying tillering has remained largely unknown, even though some genes affecting tiller initiation have been identified. More importantly, studies on the molecular mechanism underlying tillering have focused on annuals until now. Nevertheless, tillering is a more significant aspect of perennials than annuals, as yields and asexual reproduction are affected by tillering in perennial forage grasses [4, 8]. Undoubtedly, the molecular mechanisms underlying tillering in perennials is more complex than that in annuals owing to the complexity of perennial life-cycles; thus, there have been few studies examining the molecular mechanisms underlying tillering in perennials.

However, unraveling the regulatory mechanisms of tillering is vital for breeding high-yield perennial grasses. Thus, we used transcriptomic data for the first time to study the mechanisms underlying tillering in perennial orchardgrass. We demonstrate that the high-tillering genotype may be differentiated by their low expression patterns of genes involved in SL, ABA, and GA biosynthesis at the tillering stage, such as *MAX1* and *ABA2*. Furthermore, the core SL biosynthesis-associated genes in high-tillering orchardgrass were more insensitive than the low-tillering genotype to phosphorus deficiency which can lead to increases in SL biosynthesis, raising the possibility that there may be distinct SL biosynthesis way in tillering regulation in orchardgrass. Thus, our research has identified candidate genes that may regulate tillering in perennial grasses, which can be used to develop selective breeding strategies for high-yield perennial grasses and provide ideas for future research.

## Methods

### Screening and validation of high- and low-tillering orchardgrass genotypes

The tillering phenotypes of 432 orchardgrass (*Dactylis glomerata* L.) genotypes were first observed and evaluated in the field (Table S1). Among them, 30 genotypes exhibiting significant diversity in tillering number were identified for further measurement in both field and pot experiments. Besides, the ploidy of these genotypes were identified as diploids by flow cytometry referring to the method of Xu et al. [29]. Subsequently, six diploid candidates, including three high-tillering and three low-tillering genotypes were identified from field growth conditions for further validation in pot growth conditions. Finally, a high-tillering genotype (AKZ-NRGR667) and a low-tillering genotype (D20170203) were selected for further investigation through two validation tests. All field experiments were carried out in Yaan (38°8′N, 103°14′E, altitude 600–620 m, average annual temperature 16.0 °C, average annual precipitation 1015.2 mm, average annual sunshine duration 1161.5 h, average annual frost-free period 283 days) from the spring to early summer. All the validation experiments followed the principle of single variable control and random distribution of experimental units, and the original state of all materials was a single tiller. Seeds of orchardgrass AKZ-NRGR667 (Registered No. AKZ-NRGR667) were originally obtained from National Plant Germplasm System (NPGS), USA. The seeds of orchardgrass D20170203 (No. D20170203) were obtained from the Department of Grassland Science, Sichuan Agricultural University. The plants were grown in pots under natural environmental conditions in Sichuan Agricultural University, Yaan, Sichuan Province, China. All the plant materials used in this study comply with Sichuan Agricultural University and local guidelines. Field studies comply with local legislation and Convention on the Trade in Endangered Species of Wild Fauna and Flora.

### Tissue collection and RNA extraction

Plants with a single tiller were grown in silica sand with quantitative nutrient solution supplied every day. Forty-five individual plants of both AKZ-NRGR667 and D20170203 genotypes, of similar sizes, were randomly assigned to three independent groups of 15 replicates. All plants were grown in a greenhouse with a 22 °C/15 °C day/night temperature regime, a photoperiod of 14 h/10 h (day/night), and 70% relative humidity [30]. Samples from four different tissues (bud, shoot base, root, and leaf tissues) were simultaneously collected from biological replicates in a group when the bud was 0.5 cm in length [25, 31], and samples from the same group were mixed as a replicate. Samples were immediately frozen in liquid nitrogen and stored at − 80 °C until RNA extraction and subsequent transcriptome sequencing.

### RNA-seq and data analysis

Total RNA extracted from samples was used for RNA-seq library construction and sequencing. According to the manufacturer’s instructions, approximately 3 μg of RNA from each sample was used to create 23 sequencing libraries with the NEBNext® Ultra™ Directional RNA Library Prep Kit for Illumina® (NEB, California, USA) [32]. Library construction and transcriptome sequencing were conducted by the Novogene Bioinformatics Institute (Beijing, China) using the Illumina Novaseq 6000 platform (Illumina, San Diego, CA, USA). The resulting clean reads were mapped onto the orchardgrass reference genome using Hisat2 (v2.0.5), and then they were searched and annotated using BLASTx with an E-value cut-off of 1.0E^− 05^. Read counts per gene were expressed as the expected number of Fragments Per Kilobase of transcript sequence per Million base pairs sequenced (FPKM).

Differential expression analysis between the two groups was performed using the DESeq2 R package (1.16.1), and genes with an adjusted *P* < 0.05 and |log_2_ (FC)| ≥ 1, as identified by DESeq2, were assigned as differentially expressed [33]. The cluster Profiler R package was used to conduct Gene Ontology (GO) enrichment analysis of the identified differentially expressed genes (DEGs). In addition, GO terms with corrected *P* < 0.05 were considered significantly enriched among DEGs [34]. Finally, we used the cluster Profiler R package to test the statistical enrichment of DEGs among Kyoto Encyclopedia of Genes and Genomes (KEGG) pathways, using the minimum E-value as the filter parameter.

A weighted gene co-expression network analysis (WGCNA) was conducted using the WGCNA package in R (v3.3.0) [35]. We selected a total of 13,345 genes with FC ≥ 2 and FPKM>0 in at least two samples to conduct the WGCNA. A gene expression adjacency matrix was used to analyze the network topology, with soft thresholding = 18 in our analysis. The default settings for the other parameters in the R package were used. A topological overlap matrix was calculated by comparing the connectivity similarities between each pair of probes among all genes [32].

### Verification of candidate gene expression by quantitative real-time PCR

Based on current research into tillering mechanisms and our experimental results, we chose four DEGs *DWARF27* (*D27*), *9-cis-beta-carotene 9′,10′-cleaving dioxygenase* (*CCD7*), *carlactone synthase* (*CCD8*), and *more axillary branching1* (*MAX1*) from the SL biosynthesis pathway for further verification. First, we evaluated the expression of these four genes in shoot base and root tissues by quantitative real-time PCR (qRT-PCR), which aimed to verify the validity of RNA-seq results. Then, we conducted a phosphorus starvation experiment to further verify the differential expression of these genes in the SL biosynthesis pathway between the two genotypes, because phosphorus deficiency in plants can lead to in vivo increases in SL synthesis [36, 37]. In the phosphorus starvation experiment, 24 individual plants of both AKZ-NRGR667 and D20170203 genotypes, of similar sizes, were randomly assigned to eight independent groups of three replicates. Among which, four groups were used as control groups, and the other groups acted as treatment groups. All plants were grown hydroponically for a month on half-strength Hoaglands with Pi in a greenhouse with a 22 °C/15 °C day/night temperature regime, a photoperiod of 14 h/10 h (day/night), 70% relative humidity. For the Pi starvation time-course experiment, half-strength Hoagland solution without Pi was applied to treatment groups. Meanwhile, control groups were grown with half-strength Hoagland solution with Pi. The shoot bases of the high- and low-tillering genotypes were sampled at 0, 4, 8, and 12 h after the phosphorus starvation treatment, and three replicates were prepared. These samples were respectively collected during the daytime, flash-frozen in liquid nitrogen, and stored at − 80 °C until RNA isolation.

Total RNA was extracted from samples using the HiPure plant RNA Mini Kit (MAGEN, Guangzhou, China). A total of 1 μg of RNA was used as the template for reverse transcription using the MonScript™ RTIII All-in-One Mix with dsDNase (Monad, Wuhan, China). Then, qRT-PCR was conducted on the Bio-Rad CFX Connect PCR Detection System (Bio-Rad, CA, USA) using MonAmp™ SYBR Green qPCR Mix (Monad); qRT-PCR reactions were 10 μl in volume, containing 5.0 μl of SYBR Green qPCR Mix, 0.2 μl of each primer, 3.6 μl of H_2_O, and 1 μl of cDNA samples at a 4-fold dilution. *GAPDH* was used as the internal reference gene [38]. The relative gene expression levels were evaluated using the 2^-△△Ct^ method [39]. Three replicates were conducted for each experiment. All primers were designed using Primer5 (Table S2).

## Results

### Morphological analysis of high- and low-tillering orchardgrass genotypes

From our screen of 432 orchardgrass genotypes, we obtained high- and low-tillering genotypes, AKZ-NRGR667 and D20170203, respectively. In the field validation test, a single tiller of high-tillering AKZ-NRGR667 and low-tillering D20170203 formed 82 and 19 new tillers, respectively (Figure S1C). Similarly, in the pot validation test, a single tiller of AKZ-NRGR667 and D20170203 produced 57 and 13 new tillers, respectively (Figure S1F). Thus, the tiller number of high-tillering AKZ-NRGR667 and low-tillering D20170203 were significantly different in both field and pot tests (*P* < 0.01). The flow cytometry analysis showed that both AKZ-NRGR667 and D20170203 are diploid genotypes (Figure S2), indicating the difference in tillering ability was not caused by differences in ploidy, and the two genotypes were grown under the same environmental conditions, indicating the differences were not caused by environmental effects. We also found that AKZ-NRGR667 was moderate height, thin stems, and small dark green leaves (Figure S1 and S3). Additionally, AKZ-NRGR667 exhibited stronger toughness than D20170203, which is beneficial for both lodging resistance and high yield.

### Data analysis of RNA-seq

In total, 23 qualified cDNA libraries were separately constructed and subjected to RNA-seq. The quality of RNA-seq analysis depends on the quality of sequencing and the correlations among biological replicates. In our study, Q30 values exceeded 93%, the percentage of GC was greater than 50%, and error rate was only 0.03 (Table S3). Moreover, the clean reads mapped to over 70% of unique genomic locations (Table S4). The rate of clean reads mapped to exonic regions was more than 80% (Table S5). These results indicate that the sequencing quality was high. Furthermore, the FPKM values of all samples were assessed using Pearson correlation coefficients (*R*^2^) and principal component analysis (PCA), as shown in Figure S4A and B. In general, our data was thus confirmed to be reliable and could therefore be used in subsequent analyses.

### Analysis of DEGs among different tissues of the high- and low-tillering genotypes

**Fig. 1:**
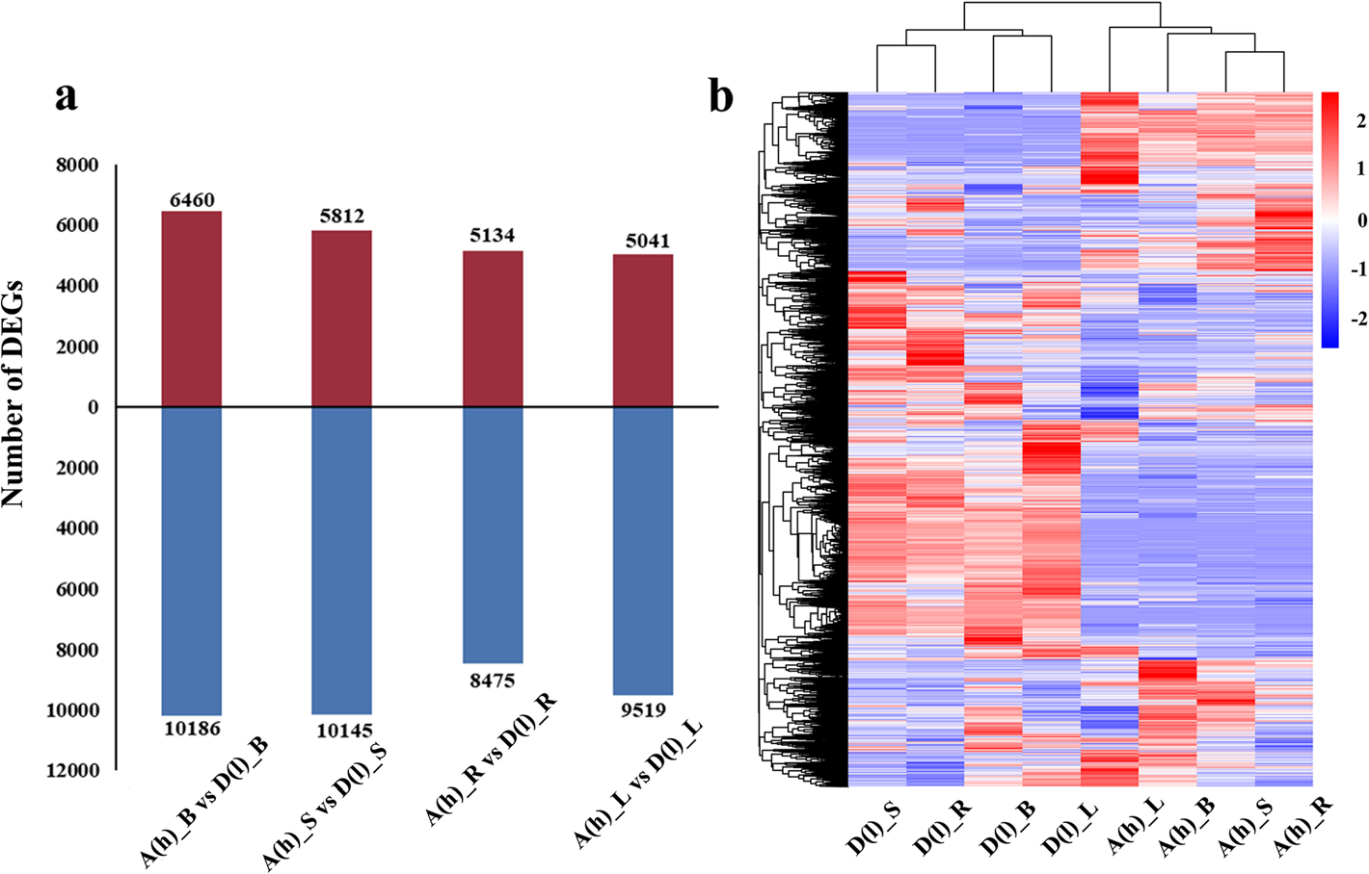
Transcriptional relationship between samples. **a**, the number of up- and down-regulated genes in four pairwise sampling stages, including A(h)_B vs D(l)_B, A(h)_S vs D(l)_S, A(h)_R vs D(l)_R, A(h)_L vs D(l)_L. **b**, the heatmap shows the respective expression levels of DEGs in each sample group, based on the average FPKM of biological replicates. The y-axis shows the cluster dendrogram of DEGs, and the X-axis shows the eight sample groups. Sample labels are as follows: A(h)_B, the tiller bud of AKZ-NRGR667; A(h)_S, the shoot base of AKZ-NRGR667; A(h)_R, the root of AKZ-NRGR667; A(h)_L, the leaf of AKZ-NRGR667; D(l)_B, the bud of D20170203; D(l)_S, the shoot base of D20170203; D(l)_R, the root of D20170203; D(l)_L, the leaf of D20170203.

To determine which DEGs were associated with tillering, four comparisons were performed between the same tissues of the two genotypes, including A(h)_B vs D(l)_B (tiller buds), A(h)_S vs D(l)_S (shoot bases), A(h)_R vs D(l)_R (roots), and A(h)_L vs D(l)_L (leaves), with thresholds of |log_2_ (FC)| ≥ 1 and *P* ≤ 0.05. Under these criteria, 16,646 (6460 upregulated and 10,186 downregulated), 15,957 (5812 upregulated and 10,145 downregulated), 13,609 (5134 upregulated and 8475 downregulated), and 14,560 (5041 upregulated and 9519 downregulated) genes were significantly differentially expressed in the A(h)_B vs D(l)_B, A(h)_S vs D(l)_S, A(h)_R vs D(l)_R, and A(h)_L vs D(l)_L comparisons, respectively (Fig. 1a). We obtained numerous DEGs in all tissues, especially in comparisons of bud and shoot base tissues, where tillering generally occurs. (Fig. 1a). Notably, the four D20170203 tissues examined had more DEGs with higher expression relative to those in AKZ-NRGR667, especially in bud and shoot base tissue comparisons (Fig. 1b).

### Functional characterization of DEGs

To examine the function of the identified DEGs, GO and KEGG pathway analyses were conducted. GO subcategory analysis revealed that “DNA integration,” “DNA metabolic process,” “ADP binding,” and “oxidoreductase activity” were significantly enriched in A(h)_B vs D(l)_B, A(h)_S vs D(l)_S, A(h)_R vs D(l)_R, and A(h)_L vs D(l)_L comparisons (Figure S5). These finding indicated that AKZ-NRGR667 and D20170203 differed in their ability to synthesize DNA and bind ADP. Moreover, KEGG analysis revealed that “Diterpenoid biosynthesis,” “Cyanoamino acid metabolism,” and “Biosynthesis of amino acids” were enriched in the A(h)_B vs D(l)_B comparison (Figure S6A). “DNA replication” was highly enriched in the A_HS vs D_LS comparison (Figure S6B), and 23 of the 27 DEGs related to DNA replication were upregulated in the A(h)_S vs D(l)_S comparison (Table S6). “Carotenoid biosynthesis” and “Biosynthesis of amino acids” were enriched in the A(h)_R vs D(l)_R comparison (Figure S6C). “Photosynthesis” was significantly enriched in the A(h)_L vs D(l)_L comparison (Figure S6D), and 26 of the 31 DEGs related to photosynthesis were upregulated in the A(h)_L vs D(l)_L comparison (Table S7).

In general, the combination of the KEGG pathway and GO subcategory analyses indicated that AKZ-NRGR667 and D20170203 differed in the expression of genes related to DNA replication and photosynthesis. Furthermore, although genes associated with “Diterpenoid biosynthesis,” “Cyanoamino acid metabolism,” “Carotenoid biosynthesis,” and “Biosynthesis of amino acids” were not significantly enriched, they could be associated with the differences in tillering ability between AKZ-NRGR667 and D20170203.

### Weighted gene co-expression network analysis

**Fig. 2:**
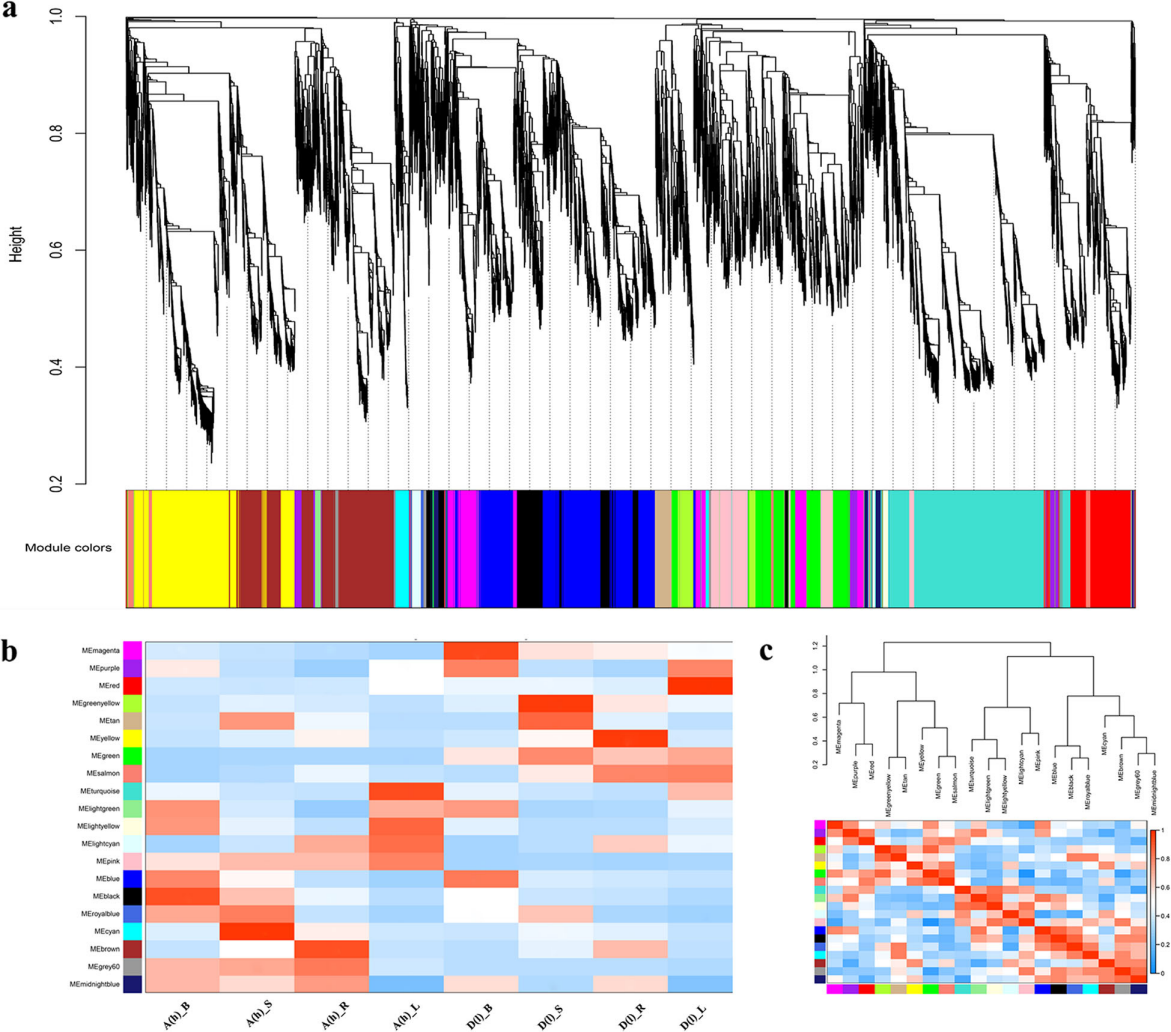
A weighted correlation network analysis of genes at eight groups. **a**, hierarchical cluster tree shows co-expression modules, identified via WGCNA. Each leaf in the tree represents one gene. Major tree branches constitute 20 modules labeled by different colors. **b**, the relationship between modules and samples. The y-axis shows the cluster module of DEGs, and the X-axis shows the eight sample groups. And the deeper red color represents the higher correlation between module and sample. The coloration scale and annotation are presented to the right of this figure. **c**, Visualization of the eigengene network represents the relationships among the modules and the clinical trait weight. Heatmap shows the correlation of different modules, and the deeper red color represents the higher correlation. The coloration scale and annotation are presented to the right of this figure

Further, WGCNA was used to investigate the co-expression network of the identified candidate genes with differential expression. According to pairwise correlations and gene expression trends among all samples, co-expression networks were constructed using normalized microarray expression data for 13,345 genes from all samples using the WCGNA R package. Among these genes shown in Fig. 2a, different colors represented each specific module, which each contain a cluster of highly correlated genes. This analysis revealed 10 distinct modules (black, brown, cyan, green, green-yellow, magenta, pink, red, turquoise, and yellow modules) with high correlation values (Fig. 2b). In particular, the pink module was only highly correlated within AKZ-NRGR667, while the green module was only highly correlated within D20170203 (Fig. 2b). Notably, half of the 10 distinct modules (brown, cyan, green-yellow, magenta, and yellow modules) are involved in phenylpropanoid biosynthesis and plant hormone signal transduction (Figure S7). The pink and green modules are both associated with ubiquitin mediated proteolysis (Figure S8A and B), which indicates that differences in ubiquitin mediated proteolysis likely differentiate AKZ-NRGR667 and D20170203. Additionally, red and turquoise modules are involved in photosynthesis (Figure S8C and D), which indicates that AKZ-NRGR667 and D20170203 likely differ in photosynthetic efficiency. We found the green-yellow module was related to plant hormone biosynthesis, including carotenoid biosynthesis and diterpenoid biosynthesis (Figure S8E), which suggests differences in hormone biosynthesis distinguish AKZ-NRGR667 and D20170203.

### Analysis of four tillering regulation pathways

**Fig. 3:**
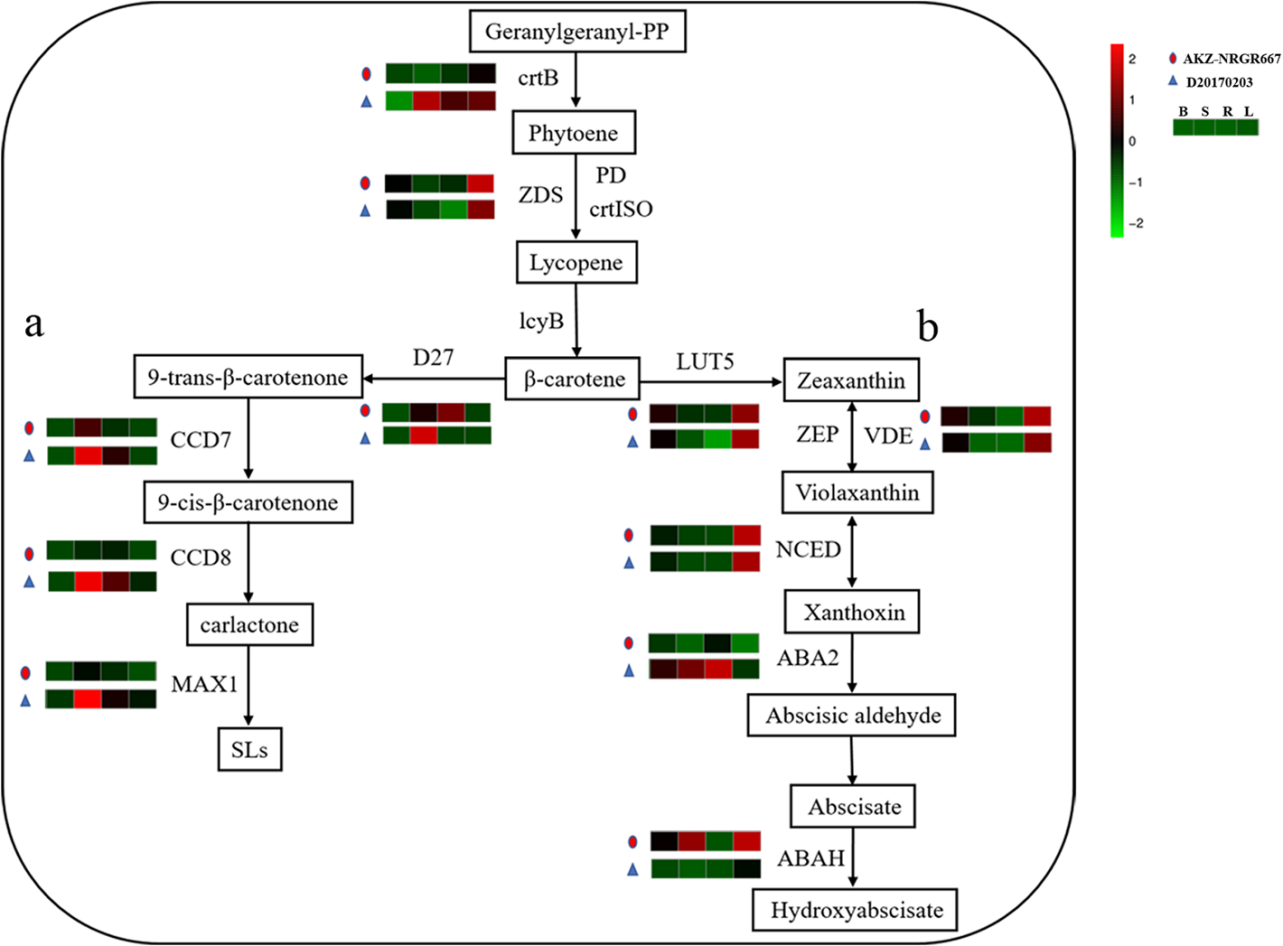
SL and ABA biosynthesis in AKZ-NRGR667 and D20170203. **a**, SL biosynthesis pathway. **b**, ABA biosynthesis pathway. The expression of genes encoding enzymes catalyzing corresponding biochemical reactions in different tissues are shown from green to red, and the coloration scale and annotation are presented to the upright corner of this figure. And B, S, R, L represent the expressions of bud, shoot base, root and leaf in the transcriptome respectively. Geranylgeranyl-PP, geranylgeranyl pyrophosphate; crtB, 15-cis-phytoene synthase; PD, 15-cis-phytoene desaturase; ZDS, zeta-carotene desaturase; crtISO, prolycopene isomerase; lcyB, lycopene beta-cyclase; LUT5, beta-ring hydroxylase; ZEP, zeaxanthin epoxidase; VDE, violaxanthin de-epoxidase; NCED, 9-cis-epoxycarotenoid dioxygenase; ABA2, xanthoxin dehydrogenase; ABAH, (+)-abscisic acid 8′-hydroxylase; D27, DWARF27; CCD7, 9-cis-beta-carotene 9′,10′-cleaving dioxygenase; CCD8, carlactone synthase; MAX1, more axillary branching1

To gain further insight into the pathway enrichment of the effect of DEGs on tillering regulation, we first compared the expression of key genes in phytohormone pathways. It has been reported that SLs and ABA can inhibit tiller formation [13]. Thus, the first and second pathways we compared were SL biosynthesis and ABA biosynthesis in carotenoid biosynthesis pathways, respectively (Fig. 3). First, geranylgeranyl pyrophosphate (GGPP) is converted into β-carotene through the action of five enzymes: 15-cis-phytoene synthase (crtB), 15-cis-phytoene desaturase (PD), zeta-carotene desaturase (ZDS), prolycopene isomerase (crtISO), and lycopene beta-cyclase (lcyB) (Fig. 3). Overall, only DEG encoding crtB was down-regulated among these DEGs encoding five enzymes in AKZ-NRGR667. Second, β-carotene was transformed into SL via four steps including four enzymes (D27, CCD7, CCD8, and MAX1) and zeaxanthin by beta-ring hydroxylase (LUT5) (Figure S9). In the SL biosynthesis pathway, four DEGs encoding these four enzymes had lower expression levels in the shoot base of AKZ-NRGR667 plants, indicating relatively reduced biosynthesis of SLs (Fig. 3a). Then, in the ABA biosynthesis pathway, zeaxanthin is converted into violaxanthin by the zeaxanthin epoxidase (ZEP) and violaxanthin is converted into zeaxanthin by violaxanthin de-epoxidase (VDE). The expression of *VDE* was higher in the shoot base of AKZ-NRGR667 relative to D20170203, indicating less violaxanthin in AKZ-NRGR667. Finally, violaxanthin is catalyzed into xanthoxin through the 9-cis-epoxycarotenoid dioxygenase (NCED). Xanthoxin is transformed into ABA through the xanthoxin dehydrogenase (ABA2) and further hydroxylated into 8′-hydroxyabscisate by the enzyme (+)-abscisic acid 8′-hydroxylase (ABAH) (Fig. 3b). In general, *ABA2* expression was downregulated, and *ABAH* expression was upregulated in AKZ-NRGR667. Taken together, these results suggest that there might be a lower accumulation of SLs and ABA in AKZ-NRGR667 relative to D20170203.

**Fig. 4:**
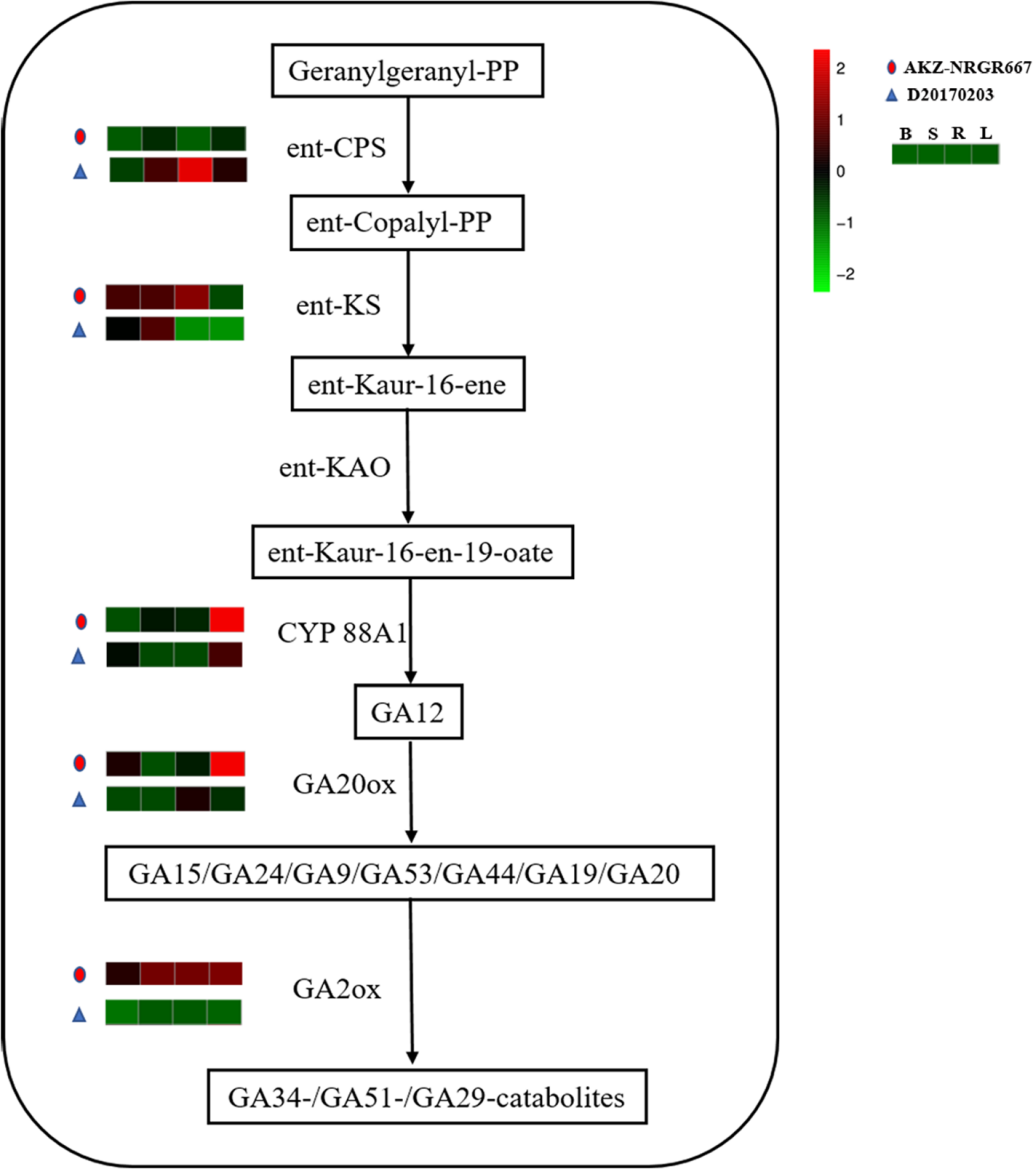
GA biosynthesis pathway in AKZ-NRGR667 and D20170203. The expression of genes encoding enzymes catalyzing corresponding biochemical reactions in different tissues are shown from green to red, and the coloration scale and annotation are presented to the upright corner of this figure. And B, S, R, L represent the expressions of bud, shoot base, root and leaf in the transcriptome respectively. Geranylgeranyl-PP, geranylgeranyl pyrophosphate; ent-Copalyl-PP, ent-Copalyl diphosphate; ent-CPS, ent-copalyl diphosphate synthase; ent-KS, ent-kaurene synthase; KAO, ent-kaurene oxidase; CYP 88A1, cytochrome P450 88A1; GA20ox, gibberellin 20 oxidase; GA2ox, gibberellin 2beta-dioxygenase

Recently, it was reported that GA triggers the degradation of SLR1, thus resulting in a decrease in tiller number [40]. Hence, the third pathway we compared was GA biosynthesis in the diterpenoid biosynthesis pathway (Fig. 4). First, GGPP is transformed into ent-copalyl-PP through catalyzation by ent-copalyl diphosphate synthase (ent-CPS). Then, ent-copalyl-PP is generated GA12 through catalysis by ent-kaurene synthase (ent-KS), ent-kaurene oxidase (KAO), and cytochrome P450 88A1 (CYP 88A1). Subsequently, GA12 is catalyzed by the gibberellin 20 oxidase (GA20ox), producing a series of GA isoforms. Among these, GA 4, GA 9, and GA 20 are all catalyzed by the enzyme GA2ox to form GA 34-, GA 51-, and GA 29-catabolites, respectively (Fig. 4). Overall, four DEGs encoding enzymes (i.e., ent-KS, CYP 88A1, GA20ox, and GA2ox) were upregulated, and 1 DEG encoding enzyme ent-CPS was downregulated in AKZ-NRGR667, implying that the biosynthesis and degradation of GAs were decreased and increased, respectively in AKZ-NRGR667 compared with D20170203. Accordingly, AKZ-NRGR667 could accumulate less GA compared with D20170203, which was mainly owing to the two DEGs encoding ent-CPS and GA2ox.

**Fig. 5:**
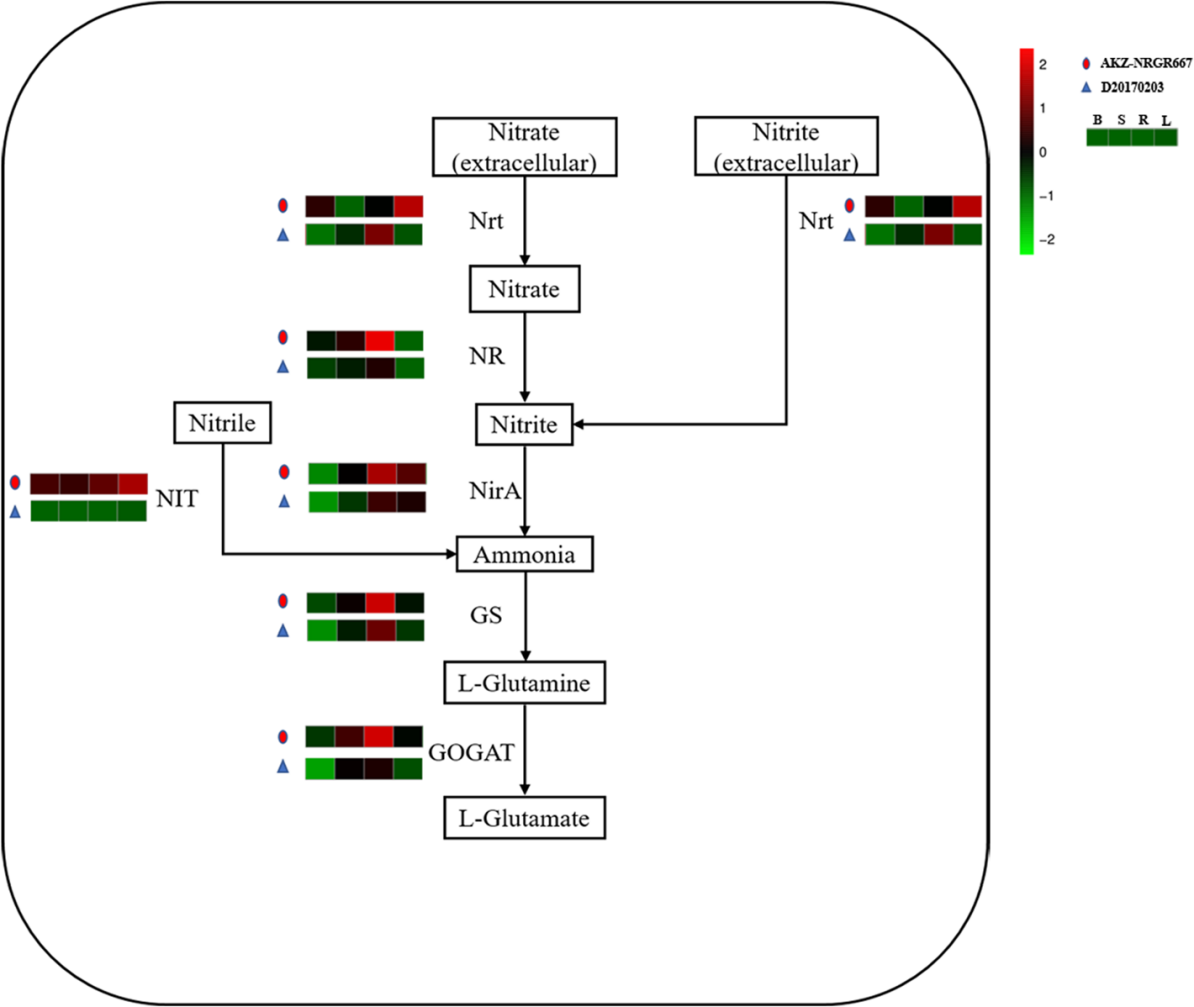
Nitrogen metabolism in AKZ-NRGR667 and D20170203. The expression of genes encoding enzymes catalyzing corresponding biochemical reactions in different tissues are shown from green to red, and the coloration scale and annotation are presented to the upright corner of this figure. And B, S, R, L represent the expressions of bud, shoot base, root and leaf in the transcriptome respectively. NRT, nitrate/nitrite transporter; NR, nitrate reductase; NirA, ferredoxin-nitrite reductase; NH_3_, ammonia; GS, glutamine synthetase; GOGAT, glutamate synthase; NIT, nitrilase; GS, glutamine synthetase

Additionally, nitrogen has a substantial regulative effect on tillering formation [10]. Thus, the fourth pathway we compared was nitrogen metabolism (Fig. 5). Extracellular nitrate or nitrite enters into the cell through the nitrate/nitrite transporter (NRT), and nitrate is converted to nitrite by nitrate reductase (NR). Then, nitrite is transformed into ammonia (NH_3_) by ferredoxin-nitrite reductase (NirA), and nitrile is transformed into NH_3_ by nitrilase (NIT). NH_3_ is converted into l-glutamine by glutamine synthetase (GS), which is catalyzed to form l-glutamate by glutamate synthase (GOGAT). *NRT*, *NR*, *NIT*, *GS*, and *GOGAT* were upregulated in tiller buds of AKZ-NRGR667 relative to those of D20170203, while only *NR* and *NIT* were upregulated in the shoot base of AKZ-NRGR667. *NR*, *NirA*, *NIT*, *GS*, and *GOGAT* were upregulated in AKZ-NRGR667 roots relative to D20170203 roots. *Nrt*, *NIT*, and *GS* were upregulated in AKZ-NRGR667 leaves relative to D20170203 leaves. Overall, the nitrogen metabolism of AKZ-NRGR667 was more active than that of D20170203, suggesting that there might be a higher utilization rate of nitrogen in AKZ-NRGR667.

### The differential expression of SL synthesis genes between AKZ-NRGR667 and D20170203 under phosphorus starvation

**Fig. 6:**
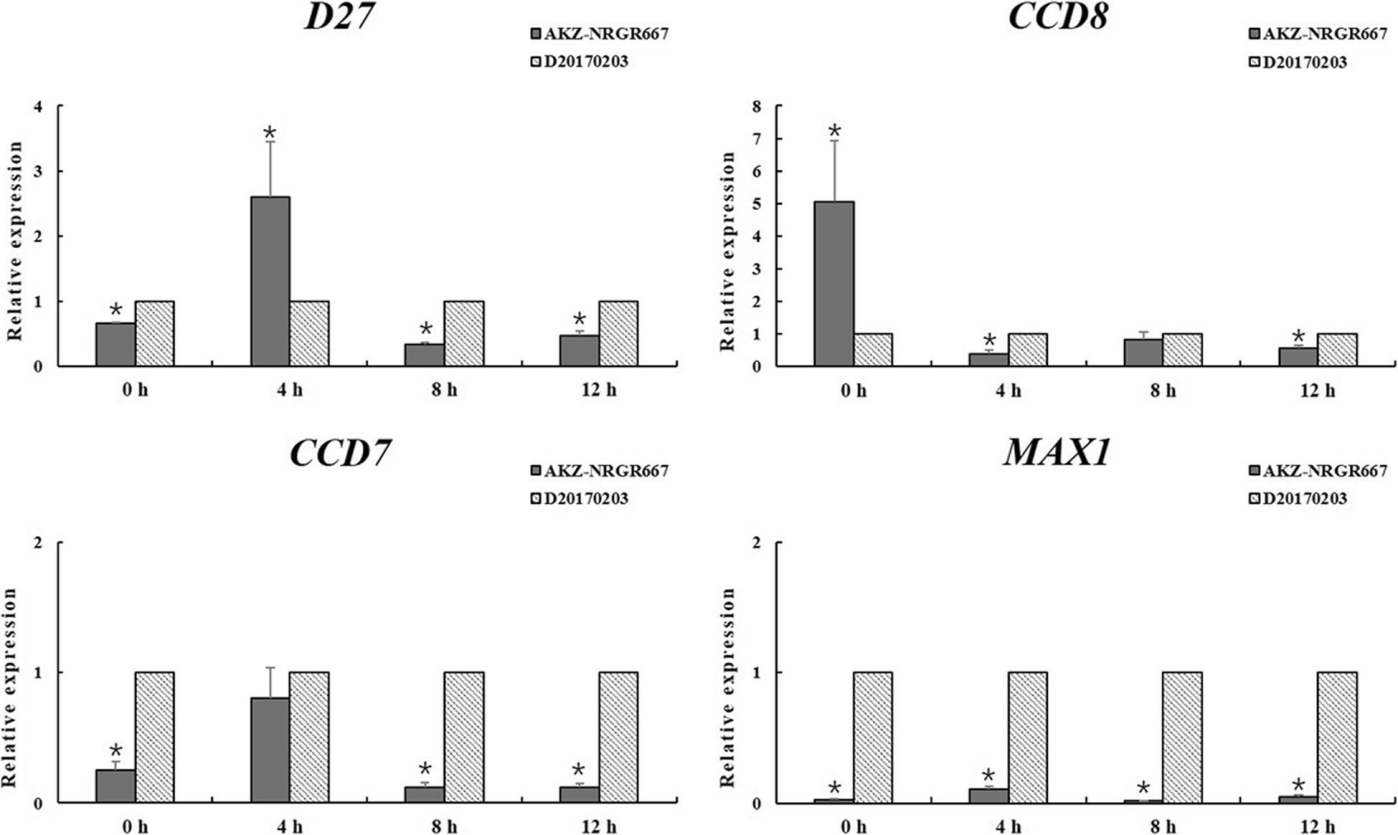
The expression level of four genes from SL biosynthesis pathway in shoot bases in phosphorus starvation experiment by qRT-PCR. *D27*, *DWARF27*; *CCD7*, *9-cis-beta-carotene 9′,10′-cleaving dioxygenase*; *CCD8*, *carlactone synthase*; *MAX1*, *more axillary branching1*. “*” indicates that *P*-value < 0.05. *GAPDH* was used as the housekeeping gene

Based on the current understanding of the molecular mechanisms underlying tillering and our results, we chose the SL biosynthesis pathway for further verification. First, the relative expression levels of four DEGs (*D27*, *CCD7*, *CCD8*, *MAX1*) from the SL biosynthesis pathway were consistent between the transcriptome results and qRT-PCR results (Figure S10). Then, we conducted a phosphorus starvation experiment to further verify the difference between AKZ-NRGR667 and D20170203 in the SL biosynthesis pathway, as phosphorus deficiencies in plants lead to increased SL synthesis in vivo [36, 37]. Under phosphorus deficiency, the relative expression levels of *CCD7* and *MAX1* from the SL biosynthesis pathway in AKZ-NRGR667 were significantly lower than those in D20170203 (*P* < 0.05). Moreover, the relative expression levels of *D27* and *CCD8* in AKZ-NRGR667 were mostly lower than those in D20170203 (Fig. 6). In general, SL biosynthesis was upregulated in D20170203 relative to AKZ-NRGR667.

## Discussion

In this work, we found that phenotypic differences between AKZ-NRGR667 and D20170203 (Figure S3) were consistent with previous studies documenting an inverse relationship between plant height and tiller number [27, 28, 41–47] and that these genotypes were also differentiated by various DEGs involved in hormone biosynthesis. The difference in tillering ability between AKZ-NRGR667 and D20170203 was not associated with differences in ploidy or environmental conditions (Figure S1 and S2). Further, the difference in tiller number between AKZ-NRGR667 and D20170203 was not caused by tiller bud dormancy, as tiller bud growth was normal between the two genotypes (data not shown). However, the photosynthetic capacity between them may differ based on differences in leaf features (Figure S3).

In the present study, approximately 26,282 DEGs were identified as differentiating tissues from the two genotypes through a detailed screening. Among the four comparisons made, most DEGs were observed in the A(h)_B vs D(l)_B and A(h)_S vs D(l)_S comparisons (Fig. 1a). This suggests that the metabolic processes in bud and shoot base tissues are more differentiated than those in the other assayed tissues. Thus, bud and shoot base tissues may be two extremely crucial tissues that are involved in regulating tiller formation. Furthermore, more of these DEGs had higher expression in the bud or shoot base tissues of D20170203 compared with AKZ-NRGR667 (Fig. 1b), which might be responsible for differential tillering phenotypes.

GO subcategory analysis revealed that AKZ-NRGR667 and D20170203 differed in their ability to synthesize DNA and bind ADP (Figure S5). KEGG pathway analysis of DEGs also revealed that the processes of DNA replication and photosynthesis were likely upregulated in AKZ-NRGR667 relative to D20170203 (Figure S6B and D). On the one hand, the dark green phenotype of AKZ-NRGR667 leaves may be related to its higher photosynthesis efficiency, which would make up for the decreased photosynthesis associated with its smaller leaf area. On the other hand, DNA replication and photosynthesis were inferred by the KEGG analysis to play an important role in AKZ-NRGR667, which is characterized by strong growth. Thus, active DNA replication and photosynthesis are two of the likely factors underlying the difference in tiller number between AKZ-NRGR667 and D20170203. In addition, some DEGs are involved in plant hormone synthesis and nitrogen metabolism (Figure S6A and C). WGCNA also revealed some modules involved in ubiquitin-mediated proteolysis, photosynthesis, nitrogen metabolism, and plant hormone biosynthesis (Figures S7 and S8). Thus, by considering the results of WGCNA and KEGG pathway together, we demonstrate that differences in the expression pattern of genes involved in SLs, ABA, GA biosynthesis differentiate the high-tillering AKZ-NRGR667 genotype and the low-tillering D20170203 genotype during the tillering stage (Figs. 3, 4 and 5). While SLs, ABA and GA play an important role in the formation of tillers [13]. Therefore, our results that genes involved in SLs, ABA, GA biosynthesis differentially expressed between the high-tillering and low-tillering genotype, indicate the differential accumulations of SLs, ABA and GA between AKZ-NRGR667 genotype and D20170203 genotype, which could be responsible for their different tillering phenotypes.

In the present work, four DEGs (*D27*, *CCD7*, *CCD8*, and *MAX1*) involved in SL biosynthesis were downregulated in the shoot base of AKZ-NRGR667 (Fig. 3a), indicating a reduction in SL biosynthesis. Therefore, the increased number of tillers in the high-tillering AKZ-NRGR667 could be a consequence of decreased SL biosynthesis, as SLs are a class of phytohormones that inhibit bud outgrowth to regulate shoot branching [15, 48–50]. In addition, the expression of *D3*, *D14*, *D53* and *MADS57* involving in SL signal transduction [28, 51], did not significantly differ between AKZ-NRGR667 and D20170203 (Table S8). Therefore, we conclude that the difference in tiller number between AKZ-NRGR667 and D20170203 may be associated with the difference in SL biosynthesis.

The low expression of ABA synthesis genes in AKZ-NRGR667 may lead to less accumulated ABA compared with D20170203 (Fig. 3b). This suggests that AKZ-NRGR667 buds grow faster than D20170203 buds because ABA inhibits the outgrowth of lateral shoots [13, 52]. However, the molecular mechanism of tillering mediated by ABA remains unclear. Although ABA levels have been reported to usually rise under increased NCED activity [53], we found that the increased ABA biosynthesis observed in D20170203 was associated with an upregulation of *ABA2* and downregulation of *ABAH*. This reflects that ABA accumulation may be controlled by different key genes in different species. Based on these results, we hypothesize that *ABA2* and *ABAH* are two key genes in the ABA biosynthesis pathway that control the outgrowth of buds in orchardgrass.

We also inferred that AKZ-NRGR667 may accumulate less GA relative to D20170203, mainly as a consequence of two DEGs encoding ent-CPS and GA2ox (Fig. 4), and this might underly the differences in tillering ability between the two genotypes. Additionally, tillering is inhibited by GA, and high tiller number can be induced by *GA2oxs* overexpression [11, 54]. Therefore, overexpression of *GA2oxs* is a clear way to reduce GA levels and improve yields by increasing tiller number in orchardgrass. GA was recently reported to trigger the degradation of SLR1, resulting in plants with fewer tillers [40]. However, *SLR1* was barely expressed in any tissues of AKZ-NRGR667 and D20170203 in our study (Table S8). Hence, we hypothesize that GA controls tillering in orchardgrass through pathways beyond the GA-SLR1 pathway.

Based on the above results, we think the tillering ability of orchardgrass is probably determined by complex regulation associated with plant hormones. ABA and GA were recently reported to enhance tiller formation by suppressing SL biosynthesis [17, 18]. However, in our results, the accumulation of GA, ABA, and SLs could be all lower in the high-tillering AKZ-NRGR667 or higher in the low-tillering D20170203. This suggests that the difference in tillering phenotype between AKZ-NRGR667 and D20170203 might not be produced by interactions between GA and SLs or ABA and SLs. Taken together, these results indicate that there might be decreased accumulation of GA, ABA, and SLs in AKZ-NRGR667 relative to D20170203, which might explain the difference in tillering ability between them.

Transcripts of genes involved in nitrogen metabolism were more abundant in AKZ-NRGR667 than in D20170203 in our study (Fig. 5). It has been reported that higher levels of nitrogen in plant tissues promote active tillering [55]. Although shoot branching is affected by various nutrient factors, including N, P, and K [56, 57], the nutrient supply in our experiment was consistent between the two genotypes. These results accordingly suggest that there might be a higher utilization ratio of nitrogen in AKZ-NRGR667, which could cause the observed high-tillering phenotype. In addition, nitrogen metabolism depends on the energy supplied by photosynthesis, which differs between AKZ-NRGR667 and D20170203 (Figure S6D). Accordingly, the different nitrogen utilization efficiencies in AKZ-NRGR667 and D20170203 could be partly caused by differences in photosynthetic efficiency (Figure S6D). Further, it was reported that energy competition between plant nitrogen and carbon metabolisms could be coordinated by increased photosynthetic efficiency [58], which suggests that AKZ-NRGR667 is better able to coordinate nitrogen and carbon metabolisms in order to sustain rapid growth because of its higher photosynthetic efficiency compared with D20170203.

In general, four pathways were identified and examined in detail, revealing that tillering in orchardgrass could be regulated by complex mechanisms mediated by plant hormones and/or nitrogen metabolism. Among these factors, we chose the SL biosynthesis pathway for further study, as tiller number can be increased by decreases in SL biosynthesis. Phosphates are a negative regulator of SL biosynthesis that can alter the expression of SL biosynthetic genes [59], and regulatory pathways related to phosphate were not significantly different our study, suggesting that the difference in tillering ability between AKZ-NRGR667 and D20170203 is not associated with phosphorus-related pathways. Therefore, we conducted two qRT-PCR experiments, under standard and phosphorus-deficient conditions, in order to further verify differences in the SL pathway between the AKZ-NRGR667 and D20170203 genotypes. First, the relative expression levels of four DEGs (*D27*, *CCD7*, *CCD8*, and *MAX1*) involved in the SL biosynthesis pathway from transcriptome results were consistent with qRT-PCR results (Figure S10). Then, we conducted a phosphorus starvation experiment to further verify the differences between AKZ-NRGR667 and D20170203 in the SL biosynthesis pathway. A previous gene expression analysis indicated that phosphate deficiency increases the transcription levels of *D27* and *CCD8* [59]. Combined with the results of our phosphorus deficiency experiment, the relative expression levels of *CCD7* and *MAX1* from the SL biosynthesis pathway in AKZ-NRGR667 were significantly lower than those in D20170203 (Fig. 6). This finding reflects the core SL biosynthesis-associated genes in the high-tillering orchardgrass being more insensitive to phosphorus deficiency than in the low-tillering genotype. Therefore, we infer that this sensitivity moderated by SL biosynthesis genes explains the difference in the number of tillers between AKZ-NRGR667 and D20170203. Moreover, SL biosynthesis in orchardgrass appears to be mainly controlled by two genes (*CCD7* and *MAX1*) according to the above results, indicating that *CCD7* and *MAX1* are two key genes that in turn regulate the process of tillering in orchardgrass via the SL pathway. However, there is little information about the key regulatory genes underlying SL biosynthesis [60]. Hence, a thorough analysis of these two SL pathway genes is still required to determine their roles in detail.

## Conclusions

In conclusion, we used transcriptomic data to study the tillering mechanism of perennial orchardgrass. We demonstrate that high-tillering genotypes may be differentiated by their low expression patterns of genes involved in SL, ABA, and GA biosynthesis at the tillering stage, such as *MAX1* and *ABA2*, and vice versa. Furthermore, the core SL biosynthesis-associated genes in high-tillering orchardgrass were more insensitive than the low-tillering genotype to phosphorus deficiency which can lead to increases in SL biosynthesis, raising the possibility that there may be distinct SL biosynthesis way in tillering regulation in orchardgrass. Our research has revealed some candidate genes involved in the regulation of tillering in perennial grasses that is available for establishment of new breeding resources for high-yield perennial grasses and will serve as a new resource for future studies into molecular mechanism of tillering regulation in orchardgrass.

## Supplementary information

**Additional file 1 **: **Supplemental Figure S1** The morphological photographs of D20170203 and AKZ-NRGR667. **A** and **B**, photographs of D20170203 and AKZ-NRGR667 in field experiment. **C**, the tiller number of D20170203 and AKZ-NRGR667 in field experiment. **D** and **E**, photographs of D20170203 and AKZ-NRGR667 in pot experiment. **F**, the tiller number of D20170203 and AKZ-NRGR667 in pot experiment. “*” indicates that *P*-value < 0.01.

**Additional file 2 **: **Supplemental Figure S2** The nuclei DNA content of D20170203 and AKZ-NRGR667 by flow cytometry analysis.

**Additional file 3 **: **Supplemental Figure S3** The phenotype characteristics of D20170203 and AKZ-NRGR667. **A**, the plant height of D20170203 and AKZ-NRGR667. **B**, the stem of D20170203 and AKZ-NRGR667. **C**, the leaves of D20170203 and AKZ-NRGR667. Sample labels are as follows: D represents the D20170203, and A represents the AKZ-NRGR667.

**Additional file 4 **: **Supplemental Figure S4** The Pearson correlation and principal component analysis (PCA) based on all expressed genes. **A,** Pearson correlation. **B,** principal component analysis (PCA). Sample labels are as follows: A(h)_B, the tiller bud of AKZ-NRGR667; A(h)_S, the shoot base of AKZ-NRGR667; A(h)_R, the root of AKZ-NRGR667; A(h)_L, the leaf of AKZ-NRGR667; D(l)_B, the bud of D20170203; D(l)_S, the shoot base of D20170203; D(l)_R, the root of D20170203; D(l)_L, the leaf of D20170203.

**Additional file 5 **: **Supplemental Figure S5** GO functional classification of DEGs in four pairwise groups. Including **A**, A(h)_B vs D(l)_B. **B**, A(h)_S vs D(l)_S. **C**, A(h)_R vs D(l)_R. **D**, A(h)_L vs D(l)_L. The greater dot represents the more DEGs. The red color indicates the smaller padj value, and the purple color indicates the bigger padj value. The coloration scale and annotation are presented to the right of this figure.

**Additional file 6 **: **Supplemental Figure S6** KEGG functional classification of DEGs in four pairwise groups. Including **A**, A(h)_B vs D(l)_B. **B**, A(h)_S vs D(l)_S. **C**, A(h)_R vs D(l)_R. **D**, A(h)_L vs D(l)_L.. The greater dot represents the more DEGs. The red color indicates the smaller padj value, and the purple color indicates the bigger padj value. The coloration scale and annotation are presented to the right of this figure.

**Additional file 7 **: **Supplemental Figure S7** KEGG functional classification of genes from different modules of WGCNA. Including brown, cyan, greenyellow, magenta and yellow modules. The greater dot represents the more DEGs. The red color indicates the smaller padj value, and the purple color indicates the bigger padj value. The coloration scale and annotation are presented to the right of this figure.

**Additional file 8 **: **Supplemental Figure S8** KEGG functional classification of genes from different modules of WGCNA. **A**, pink module. **B**, green module. **C**, red module. **D**, turquoise. **E**, greenyellow module. The greater dot represents the more DEGs. The red color indicates the smaller padj value, and the purple color indicates the bigger padj value. The coloration scale and annotation are presented to the right of this figure.

**Additional file 9 **: **Supplemental Figure S9** The results of protein sequence alignment for genes involved in SL biosynthesis in orchardgrass. D27, DWARF27; CCD7, 9-cis-beta-carotene 9′,10′-cleaving dioxygenase; CCD8, carlactone synthase; MAX1, more axillary branching1.

**Additional file 10 **: **Supplemental Figure S10** qRT-PCR validation. The expression of genes encoding enzymes catalyzing corresponding biochemical reactions in different tissues are shown from green to red, and the coloration scale and annotation are presented to the upright corner of this Fig. S and R represent the expressions of shoot base and root respectively. D27, DWARF27; CCD7, 9-cis-beta-carotene 9′,10′-cleaving dioxygenase; CCD8, carlactone synthase; MAX1, more axillary branching1.

**Additional file 11 **: **Supplemental Table 1** The data of initial field selection

**Additional file 12 **: **Supplemental Table 2** The primers information for qRT-PCR

**Additional file 13 **: **Supplemental Table 3** RNA-seq statistics

**Additional file 14 **: **Supplemental Table 4** Statistics of annotation analysis of unigenes

**Additional file 15 **: **Supplemental Table 5** Percent of genome regions

**Additional file 16 **: **Supplemental Table 6** The detail information of KEGG in A(h)_S vs D(l)_S

**Additional file 17 **: **Supplemental Table 7** The detail information of KEGG in A(h)_L vs D(l)_L

**Additional file 18 **: **Supplemental Table 8** The expression of key genes regulating tillering

## Data Availability

All the data pertaining to the present study has been included in table and/or figure form in the manuscript and authors are pleased to share analyzed/raw data and plant materials upon reasonable request. The raw RNA-seq reads have been deposited in the NCBI database (accession: SRR11362826 -SRR11362848). The plant materials were provided by Department of Grassland Science, College of Animal Science and Technology, Sichuan Agricultural University, Chengdu, China.

## Abbreviations

GA: Gibberellin
CKs: Cytokinins
ABA: Abscisic acid
SLs: Strigolactones
BRs: Brassinosteroids
MOC1: MONOCULM 1
MOC3/TAB1/SRT1: MONOCULM 3/TILLERS ABSENT 1/STERILE AND REDUCED TILLERING 1
LAX1: LAX PANICLE 1
LAX2: LAX PANICLE 2
FON1: FLORAL ORGAN NUMBER1
AM: Axillary meristem
GA2ox: Gibberellin 2beta-dioxygenase
D27: DWARF27
D53: DWARF53
D3: DWARF3
D14: DWARF14
GO: Gene Ontology
DEGs: Differentially expressed genes
WGCNA: A weighted gene co-expression network analysis
qRT-PCR: Quantitative real-time PCR
R2: Pearson correlation
PCA: Principal component analysis
GGPP: Geranylgeranyl pyrophosphate
ent-Copalyl-PP: Ent-Copalyl diphosphate
ent-CPS: Ent-copalyl diphosphate synthase
ent-KS: Ent-kaurene synthase
KAO: Ent-kaurene oxidase
CYP 88A1: Cytochrome P450 88A1
GA20ox: Gibberellin 20 oxidase
crtB: 15-cis-phytoene synthase
PD: 15-cis-phytoene desaturase
ZDS: Zeta-carotene desaturase
crtISO: Prolycopene isomerase
lcyB: Lycopene beta-cyclase
LUT5: Beta-ring hydroxylase
ZEP: Zeaxanthin epoxidase
VDE: Violaxanthin de-epoxidase
NCED: 9-cis-epoxycarotenoid dioxygenase
ABA2: Xanthoxin dehydrogenase
ABAH: (+)-abscisic acid 8′-hydroxylase
NRT: Nitrate/nitrite transporter
NR: Nitrate reductase
NH_3_: Ammonia
NirA: Ferredoxin-nitrite reductase
GS: Glutamine synthetase
GOGAT: Glutamate synthase
NIT: Nitrilase
CCD7: 9-cis-beta-carotene 9′,10′-cleaving dioxygenase
CCD8: Carlactone synthase
MAX1: More axillary branching1
