# Supplementary figures and images for: Comparative transcriptome analyses reveal different mechanism of high- and low-tillering genotypes controlling tiller growth in orchardgrass (Dactylis glomerata L.)
Source: BMC Plant Biol. 2020 Aug 5;20:369. doi: 10.1186/s12870-020-02582-2 (PMC7409468; doi:10.1186/s12870-020-02582-2)

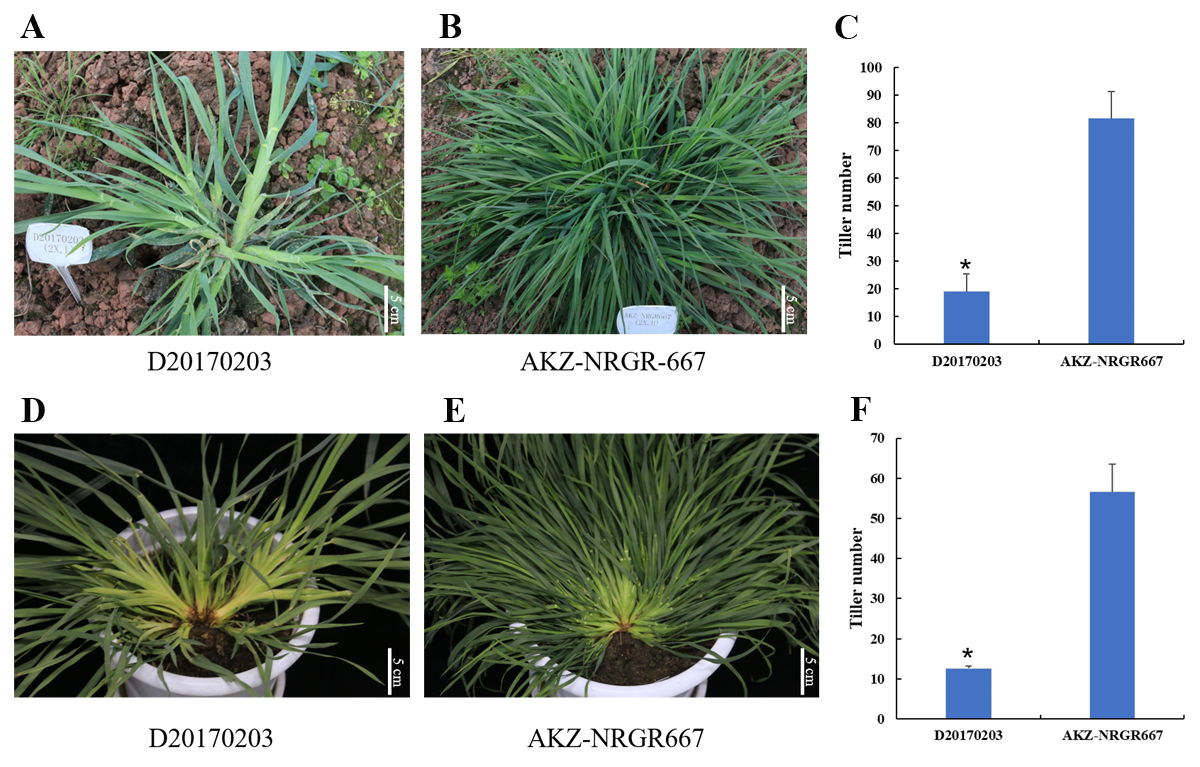

Supplement: Supplementary file 1 — Additional file 1 : Supplemental Figure S1 The morphological photographs of D20170203 and AKZ-NRGR667. A and B, photographs of D20170203 and AKZ-NRGR667 in field experiment. C, the tiller number of D20170203 and AKZ-NRGR667 in field experiment. D and E, photographs of D20170203 and AKZ-NRGR667 in pot experiment. F, the tiller number of D20170203 and AKZ-NRGR667 in pot experiment. “*” indicates that P-value < 0.01. [file 12870_2020_2582_MOESM1_ESM.png]

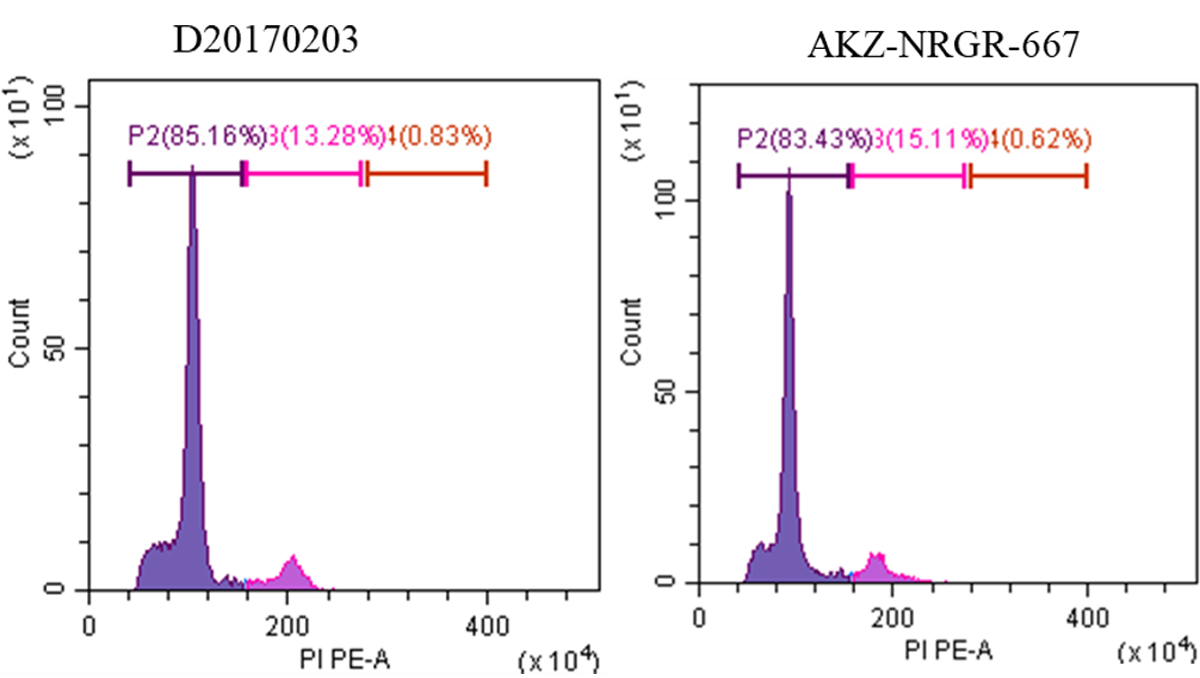

Supplement: Supplementary file 2 — Additional file 2 : Supplemental Figure S2 The nuclei DNA content of D20170203 and AKZ-NRGR667 by flow cytometry analysis. [file 12870_2020_2582_MOESM2_ESM.png]

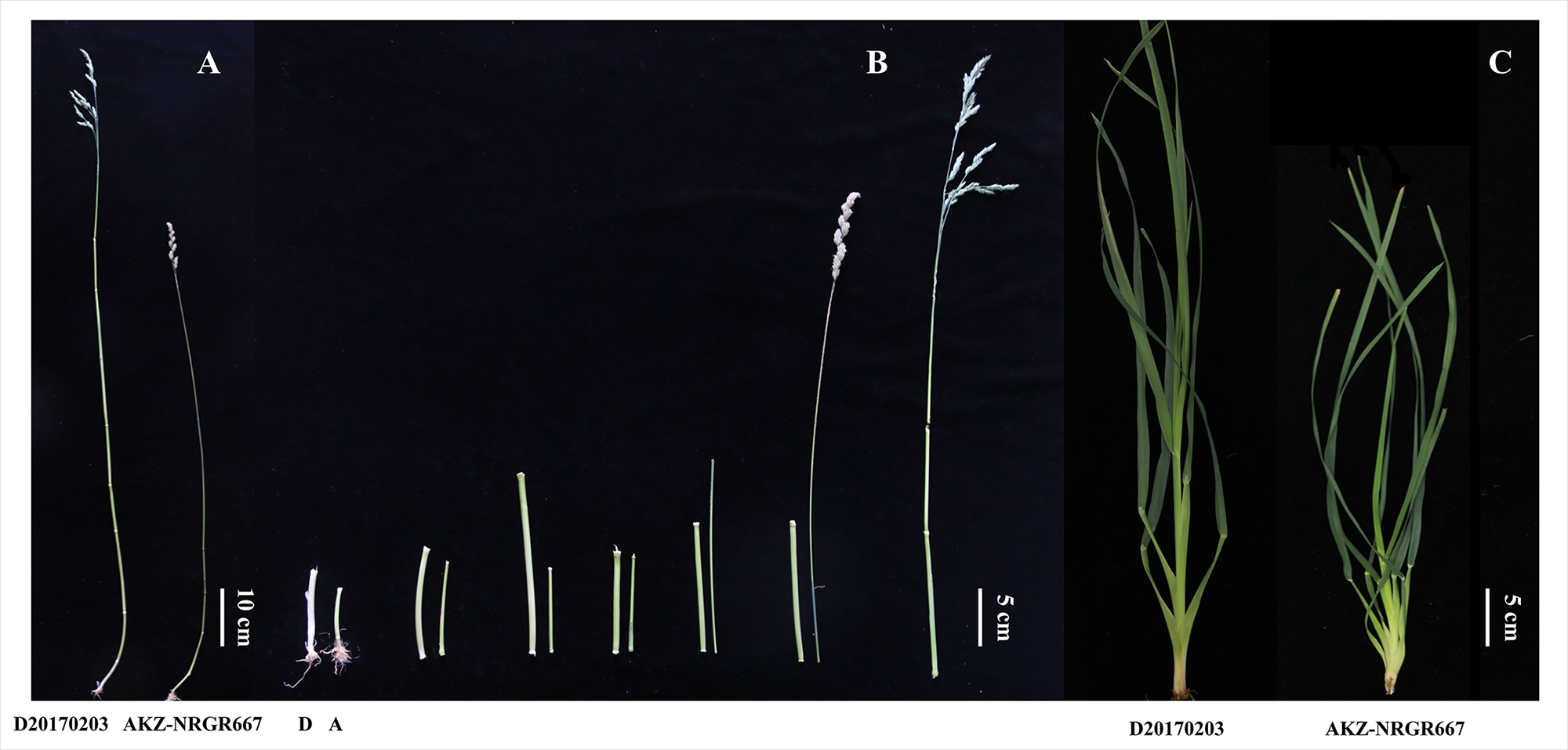

Supplement: Supplementary file 3 — Additional file 3 : Supplemental Figure S3 The phenotype characteristics of D20170203 and AKZ-NRGR667. A, the plant height of D20170203 and AKZ-NRGR667. B, the stem of D20170203 and AKZ-NRGR667. C, the leaves of D20170203 and AKZ-NRGR667. Sample labels are as follows: D represents the D20170203, and A represents the AKZ-NRGR667. [file 12870_2020_2582_MOESM3_ESM.tif]

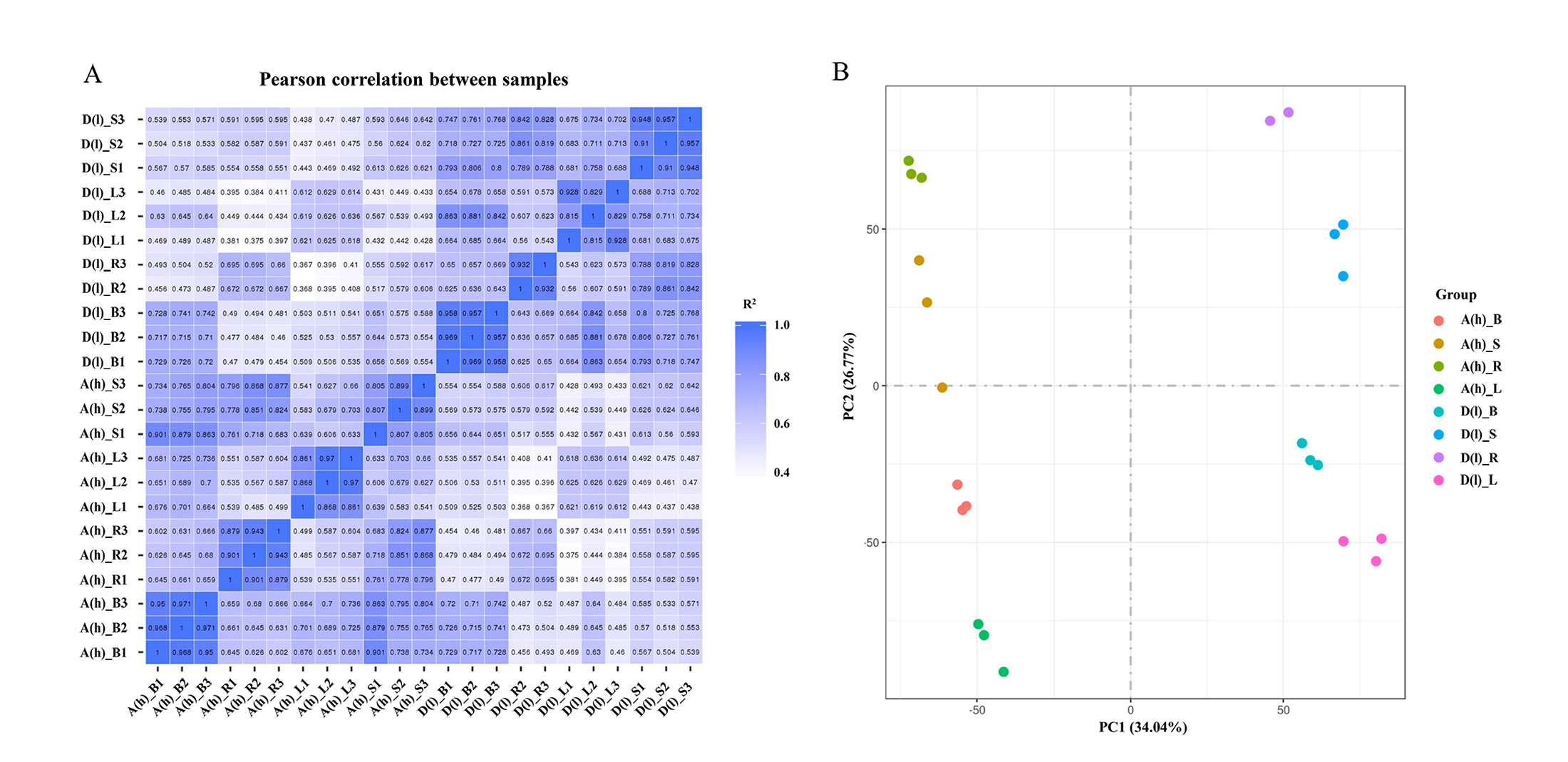

Supplement: Supplementary file 4 — Additional file 4 : Supplemental Figure S4 The Pearson correlation and principal component analysis (PCA) based on all expressed genes. A, Pearson correlation. B, principal component analysis (PCA). Sample labels are as follows: A(h)_B, the tiller bud of AKZ-NRGR667; A(h)_S, the shoot base of AKZ-NRGR667; A(h)_R, the root of AKZ-NRGR667; A(h)_L, the leaf of AKZ-NRGR667; D(l)_B, the bud of D20170203; D(l)_S, the shoot base of D20170203; D(l)_R, the root of D20170203; D(l)_L, the leaf of D20170203. [file 12870_2020_2582_MOESM4_ESM.tif]

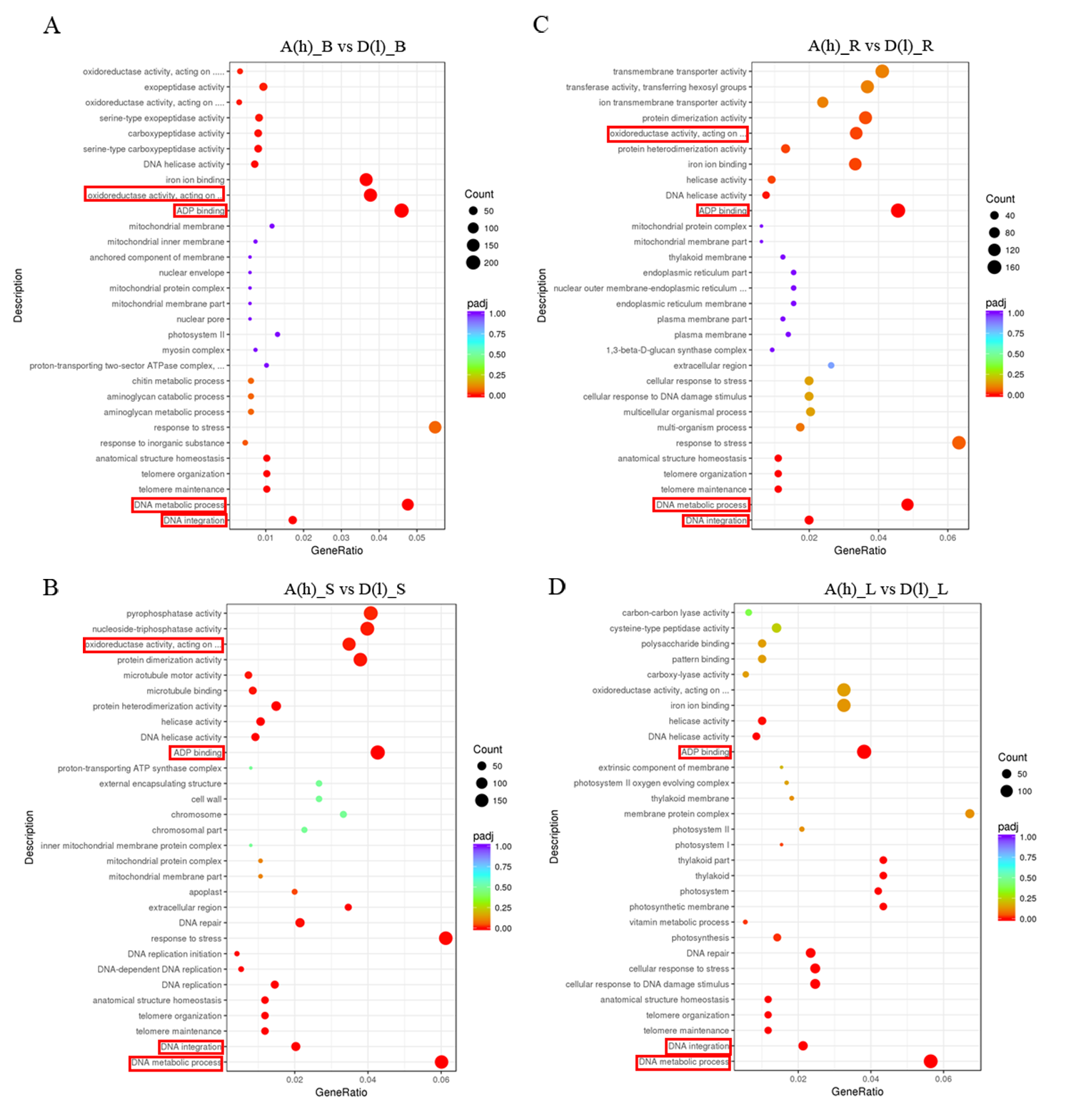

Supplement: Supplementary file 5 — Additional file 5 : Supplemental Figure S5 GO functional classification of DEGs in four pairwise groups. Including A, A(h)_B vs D(l)_B. B, A(h)_S vs D(l)_S. C, A(h)_R vs D(l)_R. D, A(h)_L vs D(l)_L. The greater dot represents the more DEGs. The red color indicates the smaller padj value, and the purple color indicates the bigger padj value. The coloration scale and annotation are presented to the right of this figure. [file 12870_2020_2582_MOESM5_ESM.tif]

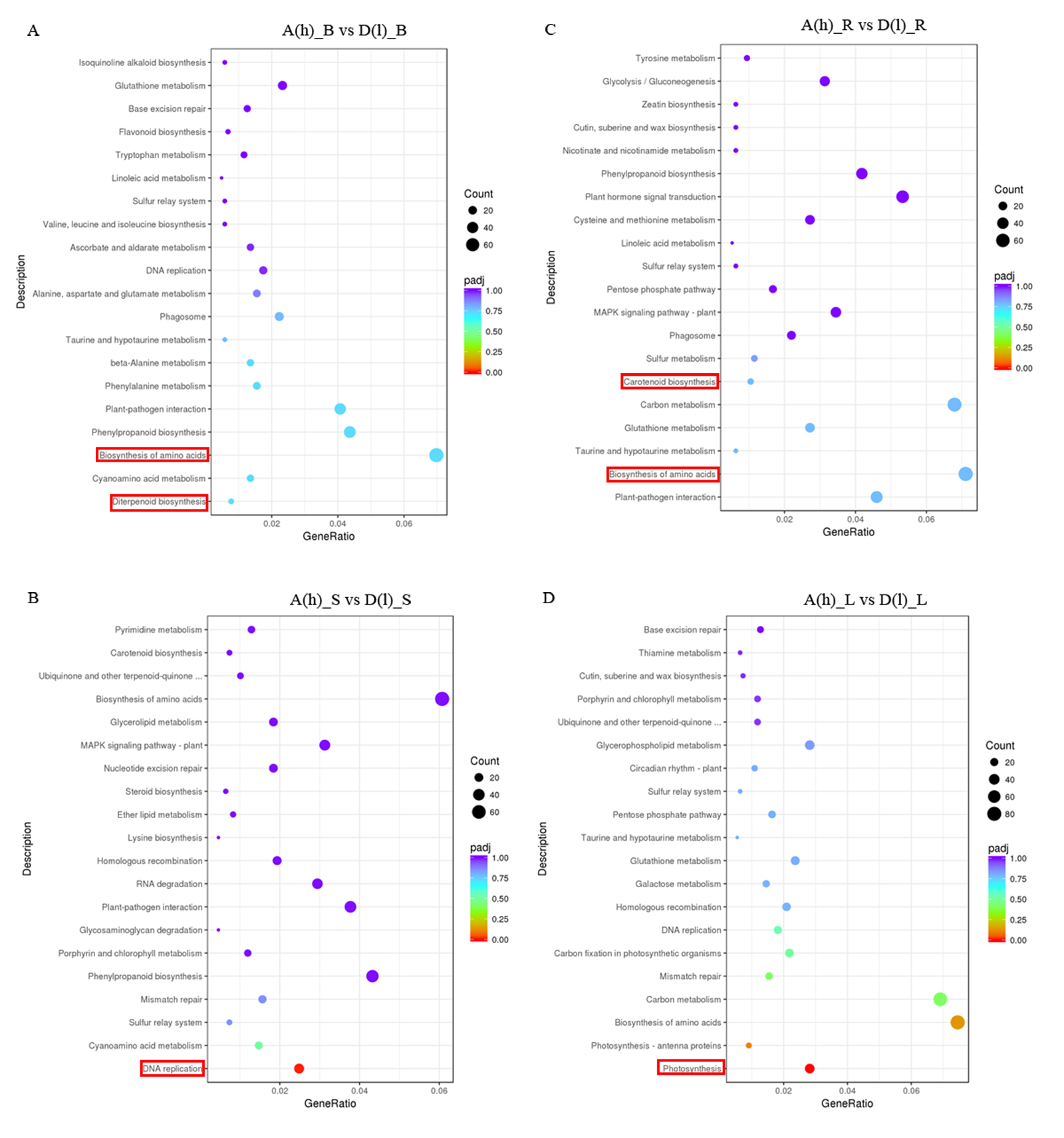

Supplement: Supplementary file 6 — Additional file 6 : Supplemental Figure S6 KEGG functional classification of DEGs in four pairwise groups. Including A, A(h)_B vs D(l)_B. B, A(h)_S vs D(l)_S. C, A(h)_R vs D(l)_R. D, A(h)_L vs D(l)_L.. The greater dot represents the more DEGs. The red color indicates the smaller padj value, and the purple color indicates the bigger padj value. The coloration scale and annotation are presented to the right of this figure. [file 12870_2020_2582_MOESM6_ESM.tif]

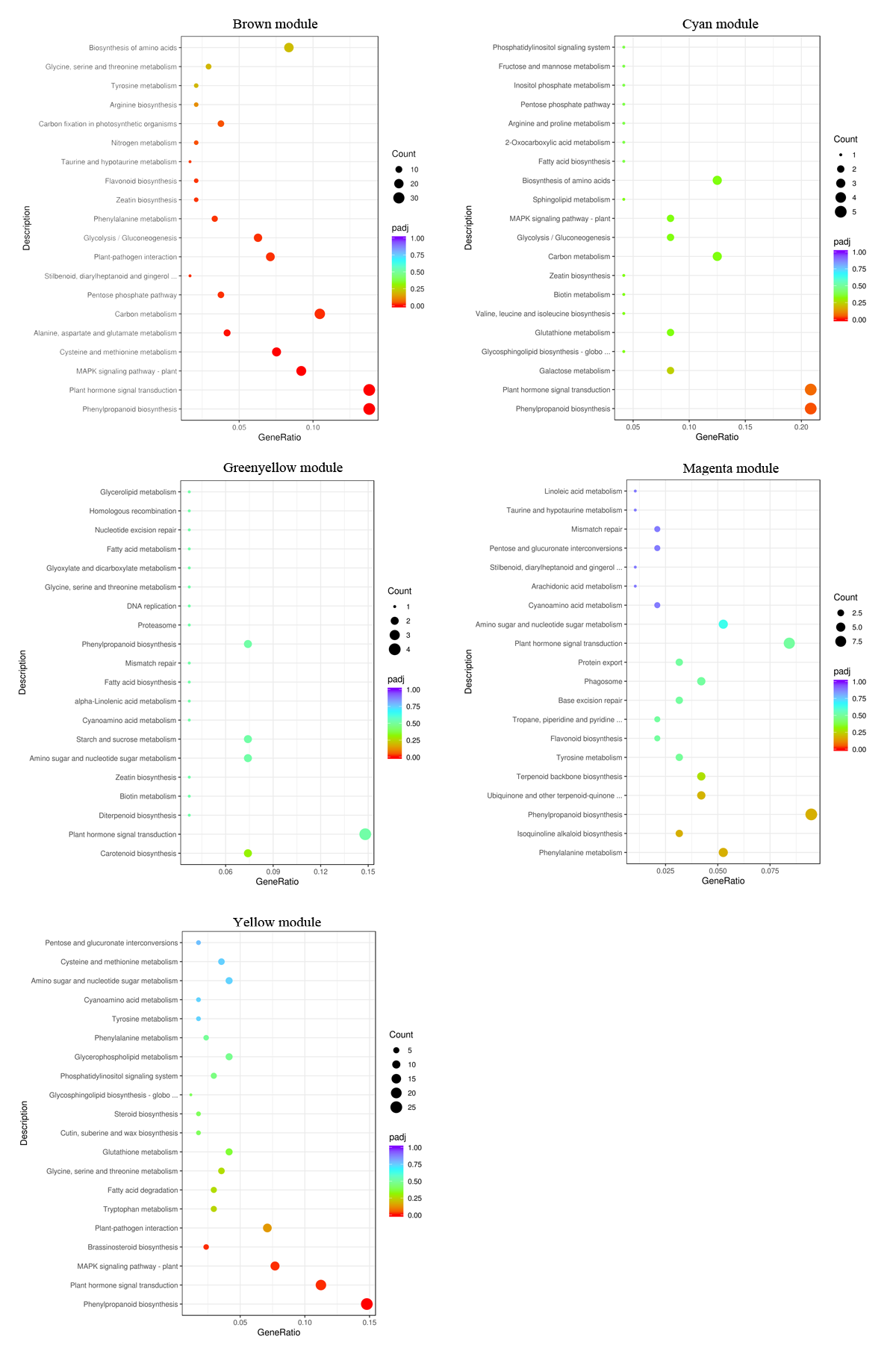

Supplement: Supplementary file 7 — Additional file 7 : Supplemental Figure S7 KEGG functional classification of genes from different modules of WGCNA. Including brown, cyan, greenyellow, magenta and yellow modules. The greater dot represents the more DEGs. The red color indicates the smaller padj value, and the purple color indicates the bigger padj value. The coloration scale and annotation are presented to the right of this figure. [file 12870_2020_2582_MOESM7_ESM.png]

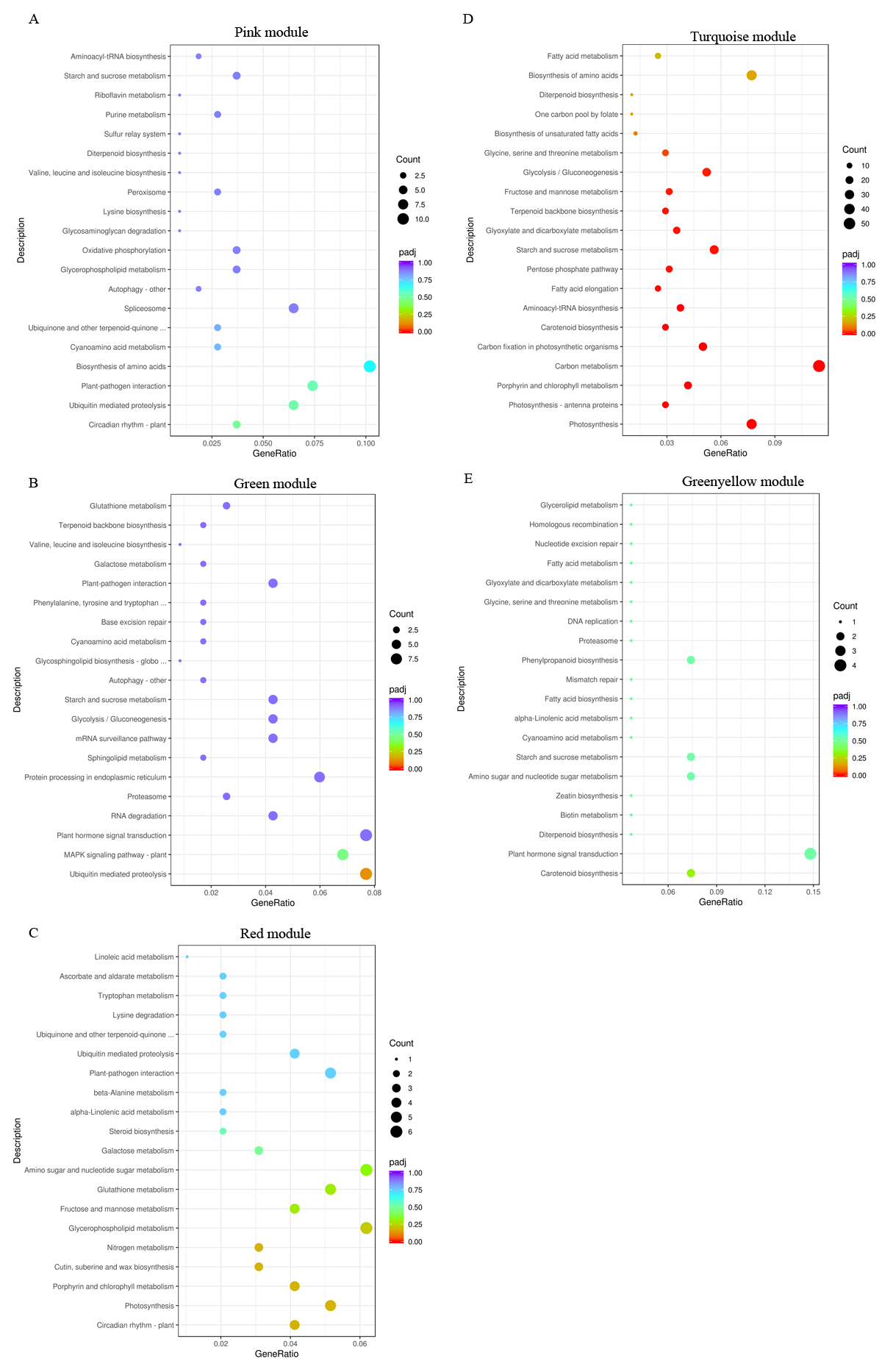

Supplement: Supplementary file 8 — Additional file 8 : Supplemental Figure S8 KEGG functional classification of genes from different modules of WGCNA. A, pink module. B, green module. C, red module. D, turquoise. E, greenyellow module. The greater dot represents the more DEGs. The red color indicates the smaller padj value, and the purple color indicates the bigger padj value. The coloration scale and annotation are presented to the right of this figure. [file 12870_2020_2582_MOESM8_ESM.png]

1. D27

1-100bp


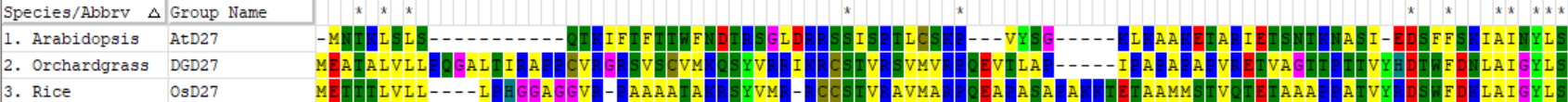


101-200bp


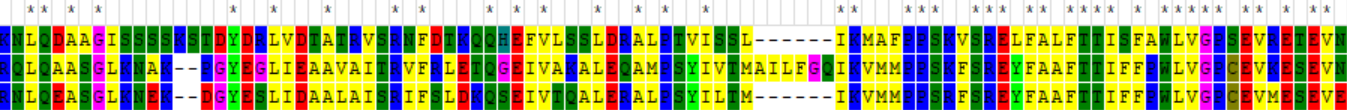


201-294bp


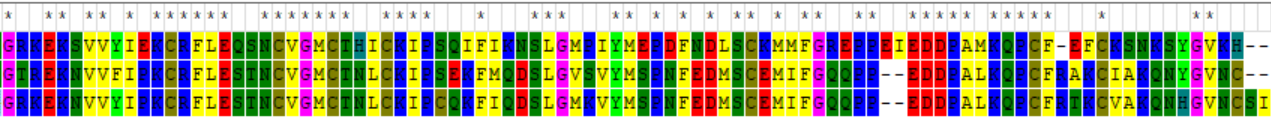


1. CCD7

1-100bp


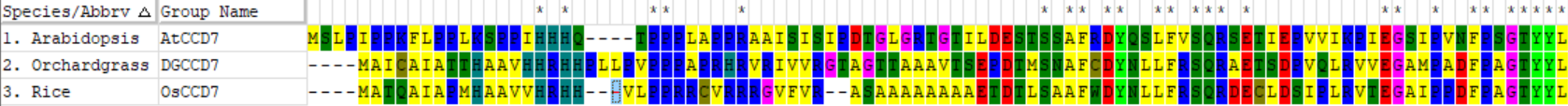


101-200bp


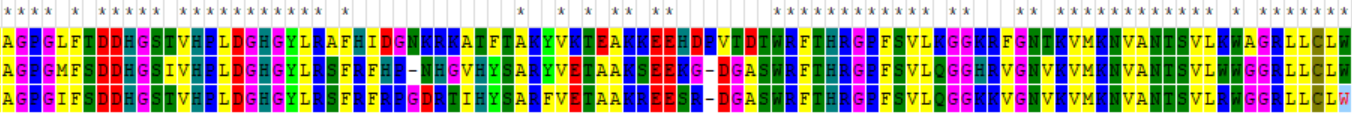


201-300bp


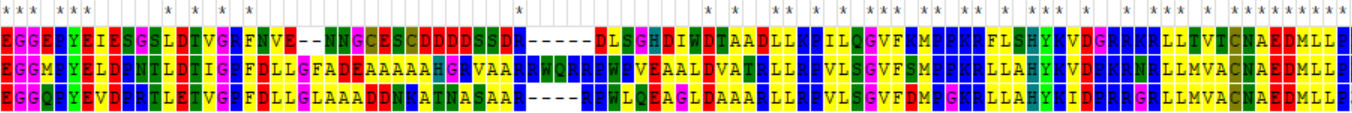


301-400bp


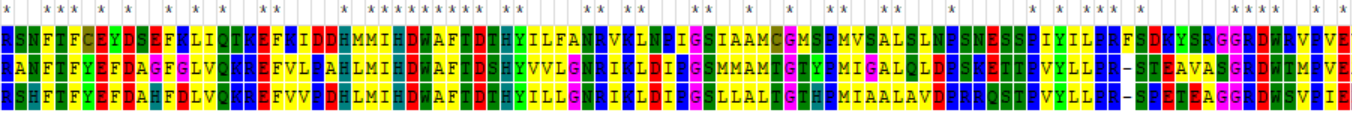


401-500bp


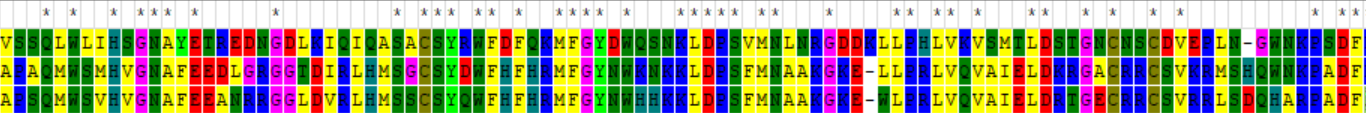


501-600bp


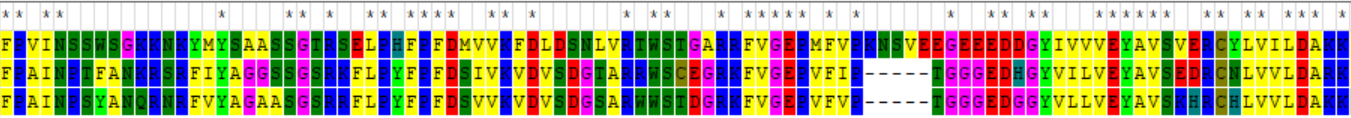


601-630bp


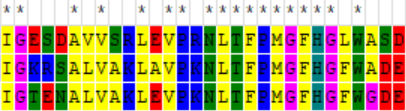


1. CCD8

1-100bp


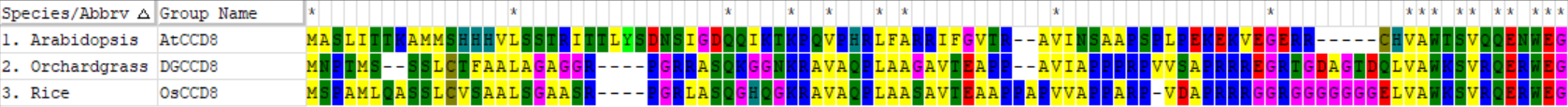


101-200bp


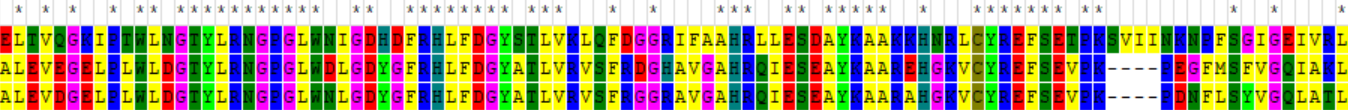


201-300bp


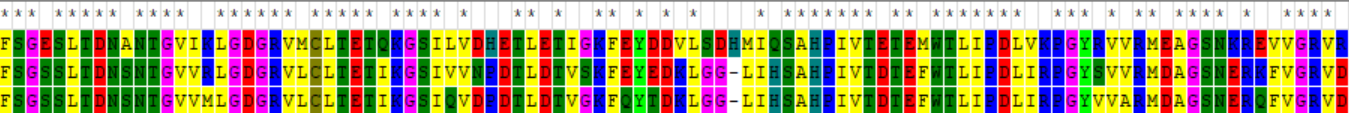


301-400bp


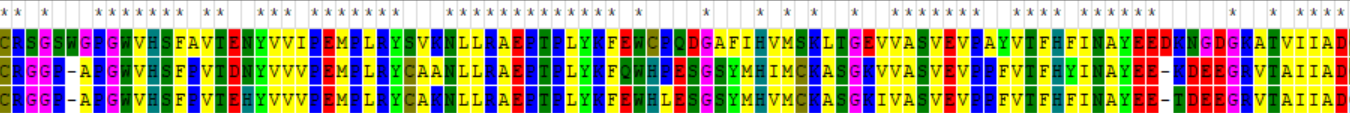


401-500bp


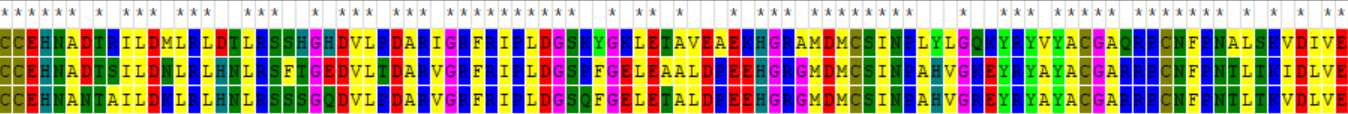


501-600bp


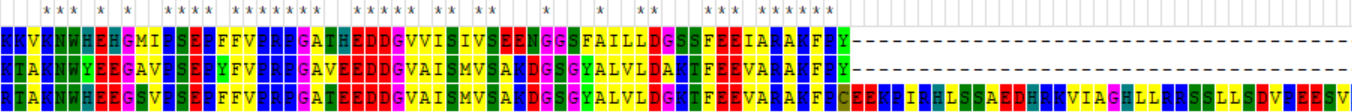


601-700bp


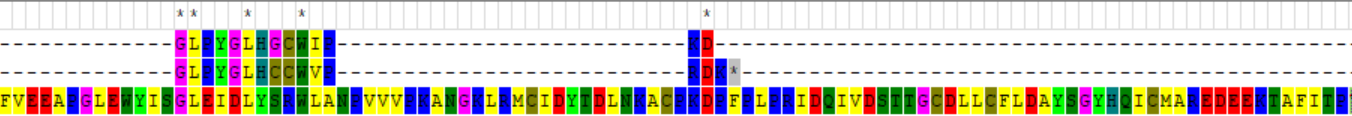


701-813bp


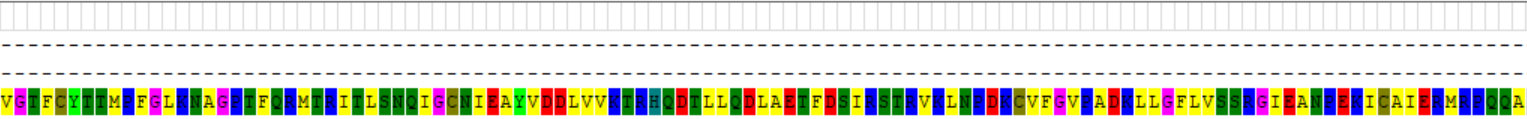


1. MAX1

1-100bp


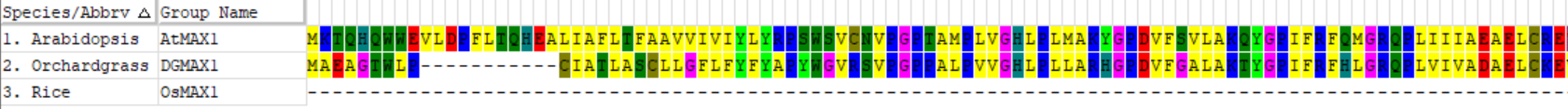


101-200bp


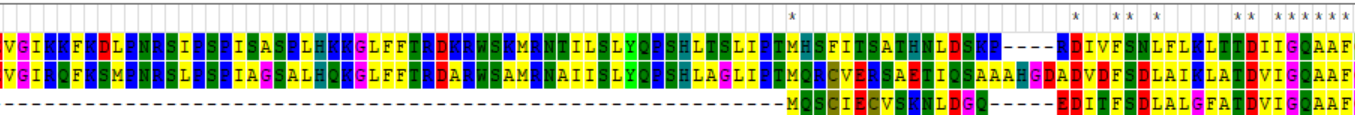


201-300bp


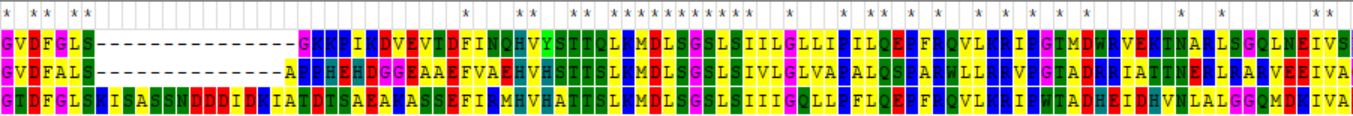


301-400bp


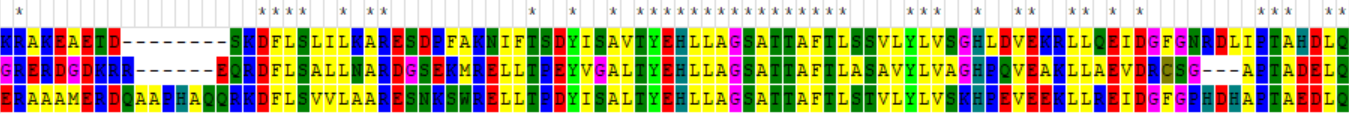


401-500bp


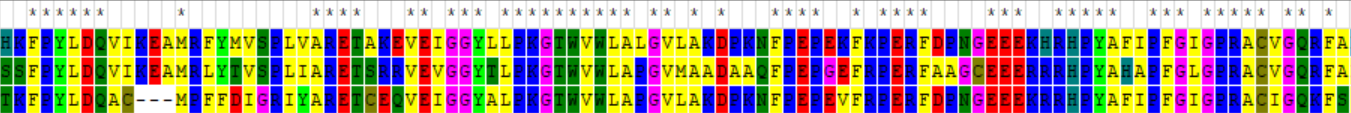


501-552bp


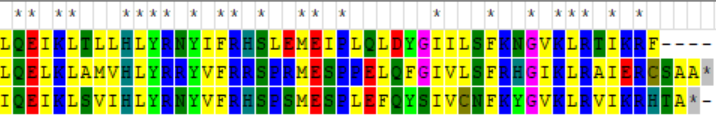

Supplement: Supplementary file 9 — Additional file 9 : Supplemental Figure S9 The results of protein sequence alignment for genes involved in SL biosynthesis in orchardgrass. D27, DWARF27; CCD7, 9-cis-beta-carotene 9′,10′-cleaving dioxygenase; CCD8, carlactone synthase; MAX1, more axillary branching1. [file 12870_2020_2582_MOESM9_ESM.docx]

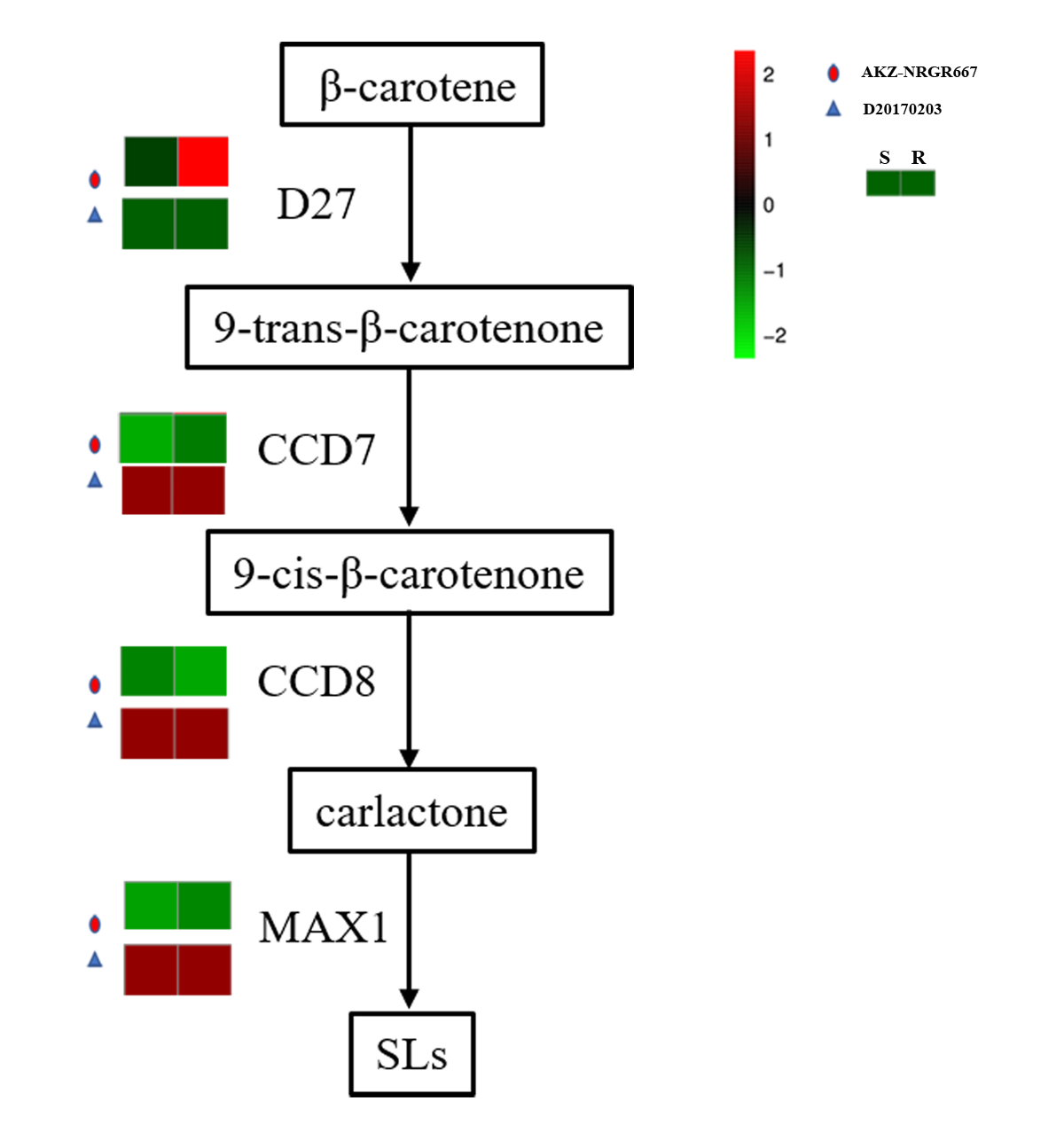

Supplement: Supplementary file 10 — Additional file 10 : Supplemental Figure S10 qRT-PCR validation. The expression of genes encoding enzymes catalyzing corresponding biochemical reactions in different tissues are shown from green to red, and the coloration scale and annotation are presented to the upright corner of this Fig. S and R represent the expressions of shoot base and root respectively. D27, DWARF27; CCD7, 9-cis-beta-carotene 9′,10′-cleaving dioxygenase; CCD8, carlactone synthase; MAX1, more axillary branching1. [file 12870_2020_2582_MOESM10_ESM.png]
